# Tissue-Specific Gain of RTK Signalling Uncovers Selective Cell Vulnerability during Embryogenesis

**DOI:** 10.1371/journal.pgen.1005533

**Published:** 2015-09-22

**Authors:** Yannan Fan, Sylvie Richelme, Emilie Avazeri, Stéphane Audebert, Françoise Helmbacher, Rosanna Dono, Flavio Maina

**Affiliations:** 1 Aix-Marseille Université, CNRS, IBDM UMR 7288, Parc Scientifique de Luminy, Case 907, Marseille, France; 2 Aix-Marseille Université UM 105, CNRS UMR7258, Inserm U1068, CRCM, Institut Paoli-Calmettes, Marseille, France; Perelman School of Medicine, University of Pennsylvania, UNITED STATES

## Abstract

The successive events that cells experience throughout development shape their intrinsic capacity to respond and integrate RTK inputs. Cellular responses to RTKs rely on different mechanisms of regulation that establish proper levels of RTK activation, define duration of RTK action, and exert quantitative/qualitative signalling outcomes. The extent to which cells are competent to deal with fluctuations in RTK signalling is incompletely understood. Here, we employ a genetic system to enhance RTK signalling in a tissue-specific manner. The chosen RTK is the hepatocyte growth factor (HGF) receptor Met, an appropriate model due to its pleiotropic requirement in distinct developmental events. Ubiquitously enhanced Met in Cre/loxP-based *Rosa26* ^*stopMet*^ knock-in context (*Del-R26* ^*Met*^) reveals that most tissues are capable of buffering enhanced Met-RTK signalling thus avoiding perturbation of developmental programs. Nevertheless, this ubiquitous increase of Met does compromise selected programs such as myoblast migration. Using cell-type specific Cre drivers, we genetically showed that altered myoblast migration results from ectopic Met expression in limb mesenchyme rather than in migrating myoblasts themselves. qRT-PCR analyses show that ectopic Met in limbs causes molecular changes such as downregulation in the expression levels of *Notum* and *Syndecan4*, two known regulators of morphogen gradients. Molecular and functional studies revealed that ectopic Met expression in limb mesenchyme does not alter HGF expression patterns and levels, but impairs HGF bioavailability. Together, our findings show that myoblasts, in which Met is endogenously expressed, are capable of buffering increased RTK levels, and identify mesenchymal cells as a cell type vulnerable to ectopic Met-RTK signalling. These results illustrate that embryonic cells are sensitive to alterations in the spatial distribution of RTK action, yet resilient to fluctuations in signalling levels of an RTK when occurring in its endogenous domain of activity.

## Author Summary

The need to achieve precise control of RTK activation is highlighted by human pathologies such as congenital malformations and cancers caused by aberrant RTK signalling. Identifying strategies to restrain RTK activity in cancer and/or to reactivate RTKs for counteracting degenerative processes is the focus of intense research efforts. We designed a genetic system to enhance RTK signalling during mouse embryogenesis in order to examine the competence of cells to deal with changes in RTK inputs. Our data reveal that most embryonic cells are capable of: 1) handling moderate perturbations in Met-RTK expression levels, 2) imposing a threshold of intracellular signalling activation despite elevated Met-RTK inputs, and/or 3) integrating variable quantitative levels of Met-RTK signalling within biological responses. Our results also establish that certain cell types, such as limb mesenchyme, are particularly vulnerable to alterations of the spatial distribution of RTK expression. The vulnerability of limb mesenchyme to enhanced Met levels is illustrated by gene expression changes, by interference with HGF chemoattractant effects, and by loss of accessibility to incoming myoblasts, leading to limb muscle defects. These findings highlight how resilience versus vulnerability to RTK fluctuation is strictly linked to cell competence and to the robustness of the developmental programs they undergo.

## Introduction

Signalling by receptor tyrosine kinases (RTKs) coordinates developmental processes and ensures tissue homeostasis. Upon ligand stimulation, RTKs activate a number of intracellular signalling cascades to influence in cells identity acquisition, movement, survival, and proliferation [1]. Cellular responses to RTKs rely on different mechanisms of regulation that establish proper levels of RTK activation, define timing of action, and exert quantitative and/or qualitative signalling outcomes. Mechanisms of RTK regulation involve transcriptional or post-transcriptional control, lateral membrane distribution, endocytosis, and intracellular compartmentalisation, for example early endosomes in which RTKs activate distinct effectors [2, 3]. The need to achieve precise control of RTK activation is highlighted by human pathologies caused by altered RTK signalling such as congenital malformations and cancer [2], and establishing strategies to restrain RTK function in disease cells is currently the focus of intense efforts [4]. Despite extensive studies, we are still far from understanding the fundamental principles of how cells perceive and integrate both quantitative and qualitative RTK signalling levels and how excess RTK signalling can perturb cellular and developmental homeostasis.

The vulnerability of cells to excess RTK signalling is conditioned by their competence to respond to an instructive RTK ligand by changing their behaviour. Such competence is influenced by the previous history of cells, during which their intrinsic capacity to respond to a given signal has been shaped through gene regulatory events leading to the acquisition or loss of molecular components necessary for the response (e.g. receptors, co-receptors, signalling modules, and transcription factors). Cellular responses to a given RTK are also conditioned by the simultaneous activation of other signalling inputs and by their synergistic or antagonistic nature [5, 6]. Vulnerability of cells to an excess of a given RTK may also vary according to whether such excess results in an enhanced activation over endogenous levels, or whether this excess would result in ectopic activation in cells that would not normally express the RTK. Thus, cell vulnerability is most likely determined by the relative contribution of all these aspects and by the intensity of RTK signalling fluctuation.

As highlighted by studies in cellular and animal models, constitutive activation of RTKs results in altered duration/intensity of intracellular signalling and/or participation of additional pathways not recruited under physiological conditions. Unsurprisingly, molecular pathways activated by aberrant RTKs are integrated within other signalling networks through crosstalk, causing signalling perturbations that in turn impact cell behaviour [7]. However, most of these studies have involved models in which enhanced activation of RTKs is achieved through strategies such as activating point mutations, oncogenic translocation, and gene amplification. These strategies lead to constitutive activated forms of RTKs that escape regulatory mechanisms, which normally modulate signalling intensity and duration. These strategies are therefore inappropriate to assess whether cells are capable of: a) buffering subtle fluctuations in RTK activation over physiological levels, and b) imposing a threshold of intracellular signalling activation despites the level of RTK input.

In order to provide insights into the capacity of cells to integrate enhanced RTK signalling above endogenous levels within developmental programs, we have engineered the first mouse model in which RTK signalling can be conditionally enhanced in a tissue-specific manner. Importantly, we opted for the wild-type version of the RTK to avoid perturbation of cellular mechanisms regulating signalling maintenance, extinction, and intracellular location. The chosen RTK is the hepatocyte growth factor (HGF) receptor Met, an appropriate model due to: a) its pleiotropic properties elicited in distinct cell types [8]; b) the variety of biological response regulated in different developmental programs and tissue homeostasis [9–11]; c) its impact on tumour evolution and resistance to anticancer therapies [8, 12–14]. During embryogenesis, alteration of the HGF/Met system in mice causes liver, placenta, muscle, and neuronal defects [15–23]. Development of muscle in the limb represents a biological model of crosstalk between limb mesenchyme and myoblasts that migrate from the somites to colonize the limb buds. Whereas migrating myoblasts express Met, limb mesenchymal cells produce the HGF acting as a chemoattractant for incoming myoblasts [15, 18, 23]. Acquisition of motility by myoblast at limb levels is strictly dependent on HGF/Met, with no other redundant molecules capable of bypassing loss of Met signalling.

To achieve conditional gain of wild-type Met-RTK signalling above an endogenous level, we employed the Cre-loxP strategy [24]. In particular, we generated *Rosa26* ^*LacZ-stop-Met*^ (*R26* ^*stopMet*^) transgenic mice that permit, upon Cre-mediated excision, to: 1) increase Met expression above endogenous levels and/or 2) impose Met signalling in cells that do not normally express Met [24]. By using the *R26* ^*stopMet*^ transgenic mice, we previously demonstrated that enhancing Met signalling in the neural lineage delays the onset of motor neuron degeneration in a murine model of amyotrophic lateral sclerosis [24]. Moreover, these mice revealed the levels of HGF required for retinal ganglion cells survival and axonal growth after optic nerve axotomy lesion [25]. In the present study, we combined *R26* ^*stopMet*^ transgenic mice with either ubiquitous or tissue-specific-Cre transgenic lines to determine which cells are vulnerable to moderately enhanced Met-RTK expression levels during development. These genetic studies revealed that exogenous Met phosphorylation is restricted to few territories in developing embryos, thus highlighting the existence of a map of competence to Met activation. Intriguingly, we found that most cells, whether relying on endogenous Met signalling or being normally devoid of Met expression, can buffer a moderate increase in RTK signalling. This correlates with a limited propagation of the enhanced Met-RTK input to its downstream signals. This demonstrates that the signalling network is resilient to intensity changes in Met-RTK input for normal development. However, one cell type, limb mesenchyme, is vulnerable to ectopic Met expression, as evidenced by a drastic impairment of muscle morphogenesis, and by molecular changes in the expression levels of some extracellular regulators of morphogens. Ectopic expression of Met in mesenchymal cells, although not interfering with early limb patterning events, prevents myoblast migration in a non-cell autonomous manner, leading to a drastic reduction of limb muscles. In contrast, enhanced Met signalling in myogenic cells themselves does not interfere with their migration or with limb muscle development. Vulnerability of limb mesenchyme is due to altered HGF bioavailability in the limbs, as assessed by functional studies. Together, our findings show that the accomplishment of developmental programs in a genetic setting with enhanced Met levels is ensured by the robustness of cell competence to buffer increased RTK activity and to conserve functional downstream signalling. These findings also indicate that developmental programs are sensitive to alterations in the spatial distribution of RTK action.

## Results

### Modelling enhanced wild-type Met signalling during mouse embryogenesis

We have previously reported the generation of the *Rosa26* ^*LacZ-stop-Met*^ (*R26* ^*stopMet*^) mice, which allow Met levels to be enhanced in a temporal and spatial regulated manner, and have used them to demonstrate that boosting neuronal RTK signalling delays disease onset in an amyotrophic lateral sclerosis animal model [24]. In the present study, we used the *R26* ^*stopMet*^ mice to explore genetically the robustness of developmental programs following enhanced wild-type RTK expression. To enhance Met-RTK signalling levels in all developing tissues, we crossed *R26* ^*stopMet*^ mice with *Tg(CMV-cre)1Cgn/J* transgenics (namely *Deleter-Cre*) [26] thus obtaining *Deleter-Cre;R26* ^*Met*^ mice/embryos (referred to as *Del-R26* ^*Met*^) in order to excise the LacZ-stop cassette in every cells and allow ubiquitous expression of the Met transgene (Met^tg^; S1A Fig). β-galactosidase staining on whole mount or section of E10.5 embryos, used as readout of absence of recombination, showed a spectrum of recombination in *Del-R26* ^*Met*^ embryos ranging from cases with only residual staining compared to *R26* ^*stopMet*^ controls (defined as fully recombined *Del-R26* ^*Met*^ embryos) to cases with a mosaic and more pronounced staining (defined as partially recombined *Del-R26* ^*Met*^ embryos; S1B–S1D Fig). These findings show that the *Deleter-Cre* line permits excision of the LacZ-stop cassette in all embryonic cell types, although with a variable efficiency. This mosaicism is in part due to the random X-chromosome inactivation in females, as the *Cre* transgene is X-linked [26]. In the present studies we only used fully recombined *Del-R26* ^*Met*^ embryos. Whole mount in situ hybridization (ISH) with a human *Met* probe, distinguishing the Met^tg^ from the endogenous mouse Met (mMet), showed ubiquitous expression of Met^tg^ in fully recombined *Del-R26* ^*Met*^ embryos compared to controls (S2 Fig). To further validate that this approach leads to enhanced Met signalling, we investigated the extent of Met activation in developmental tissues by following its phosphorylation state using anti-phospho-Met and anti-human Met antibodies in immunohistochemistry (IHC). Whereas Met^tg^ appeared to be uniformly expressed in most organs in *Del-R26* ^*Met*^ embryos, phospho-Met staining was detected in restricted cell types (S3 Fig). These findings indicate the existence of a restricted “competence map” for Met activation.

**Fig 1:**
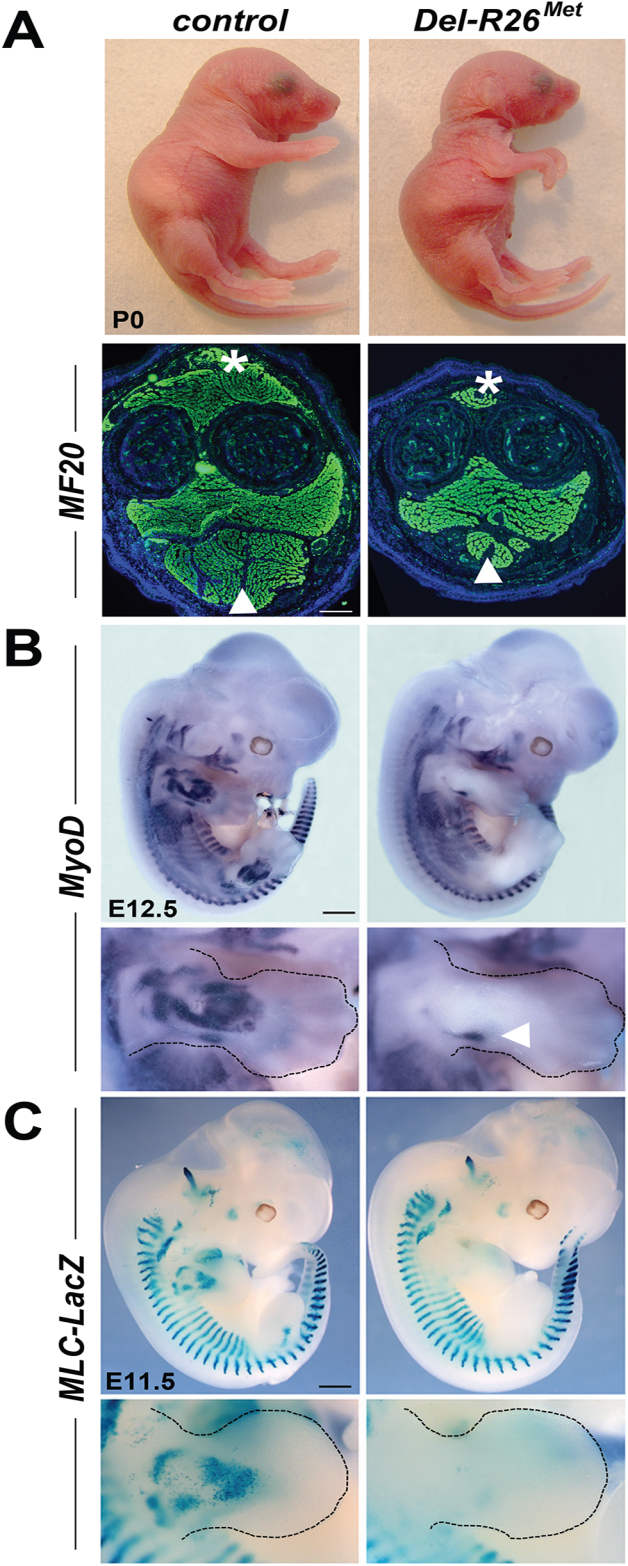
Ubiquitously excess wild-type Met in developing embryos results into hyperflexed forelimbs. (A) Top: *Del-R26* ^*Met*^ and control P0 mice showing hyperflexed limbs in mutants. Bottom: anti-myosin heavy chain II IHC using MF20 antibodies on forelimb transversal sections of P0 *Del-R26* ^*Met*^ and control mice at the level of the forearm showing almost absence of extensor (asterisk) and a great reduction of flexor (arrowhead) muscle mass in mutants. (B, C) Whole mount ISH with *MyoD* probe of E12.5 embryos (B) and β-galactosidase staining of E11.5 embryos (C) showing that developing appendicular muscles are reduced in *Del-R26* ^*Met*^ embryos (limbs are outlined in panels). The arrowhead in bottom panel B indicates developing ventral limb muscles (flexor). Scale: 500μm.

Highly recombined *Del-R26* ^*Met*^ neonates were identifiable at birth because of their hyperflexed forelimbs and weak hindlimbs (Fig 1A). Forelimb sections stained with MF20 antibodies, which recognise the myosin heavy chain II, revealed that *Del-R26* ^*Met*^ limbs were almost devoid of extensor muscles on their dorsal side, and exhibited a strong reduction of flexor muscles in the ventral side, compared to controls (Fig 1A). This unbalance between antagonist muscles leads to a pronounced wrist flexure. Whole mount ISH with a *MyoD* probe at E12.5 revealed a strong reduction of dorsal limb forming muscles in *Del-R26* ^*Met*^ mutants compared to controls, whereas other sites of *MyoD* expression were normal (Fig 1B). Next, we crossed *Del-R26* ^*Met*^ mice with the *Mlc3f-2E-nlacZ* transgenic mouse line, in which reporter gene expression is driven by the murine *Mlc3f* promoter and by the *Mlc1f/3f-3’* skeletal muscle enhancer [27]. In this mouse line, *nLacZ* (encoding nuclear β-galactosidase) is expressed in all skeletal muscles throughout development, permitting visualisation of muscle formation in fully recombined *Del-R26* ^*Met*^ embryos that no longer express the LacZ-stop cassette. Whole mount staining showed that β-galactosidase activity was drastically reduced in *Del-R26* ^*Met*^ developing limbs compared to controls (Figs 1C and S4).

**Fig 2:**
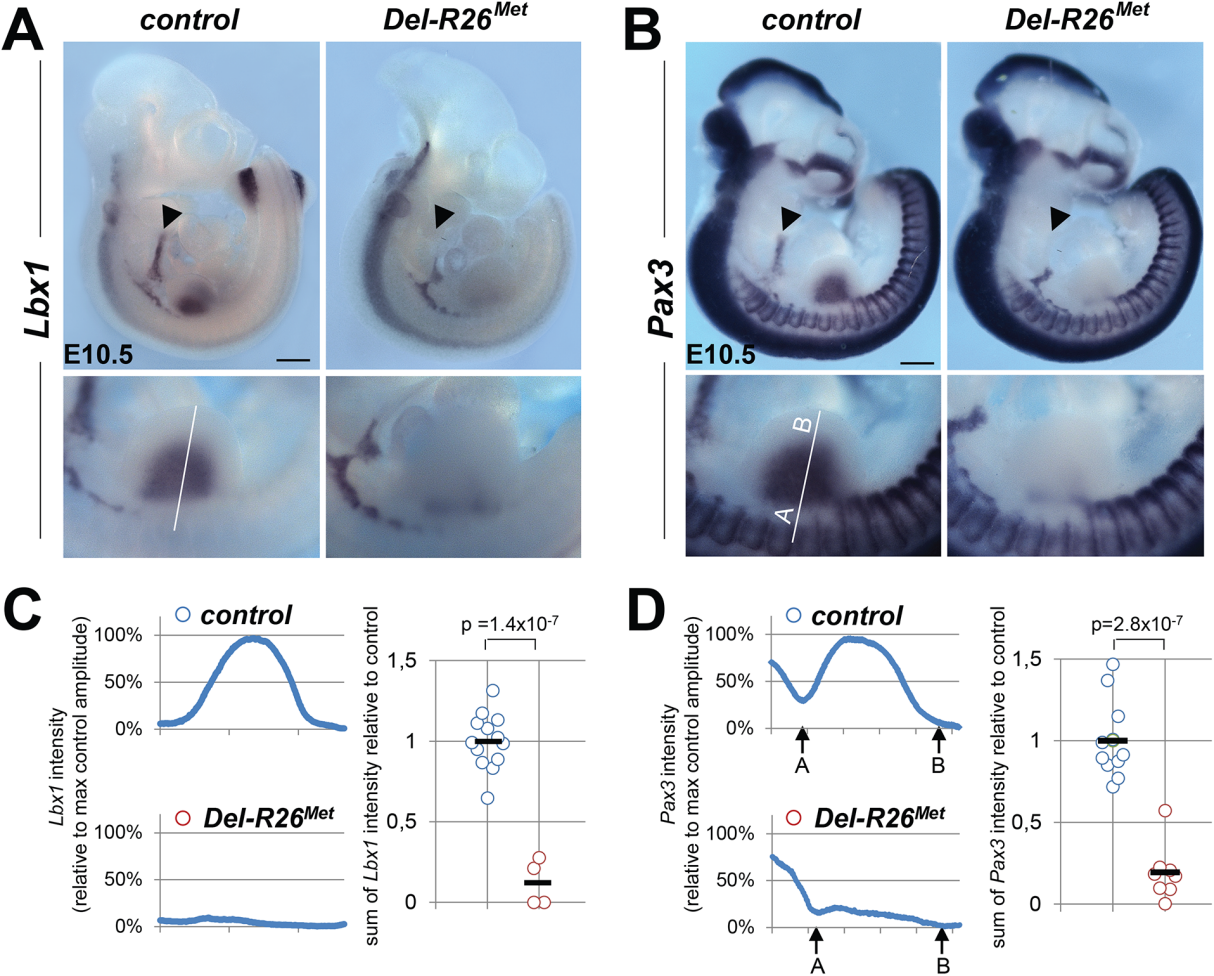
Myoblast migration is impaired in *Del-R26* ^*Met*^ mutants. (A, B) Whole mount ISH of E10.5 embryos with *Lbx1* (A) and *Pax3* (B) probes showing drastic reduction of migrating myoblasts towards the developing tongue (arrowhead), fore and hind limbs. Bottom panel reports an enlargement at forelimb levels. (C, D) Quantification analyses of *Lbx1* (C) and *Pax3* (D) positive domains in forelimbs. Left panels: each plot represents the average signal distribution along the white line in forelimbs. Right panels: quantifications and statistical analyses of the sum of signal intensity based on intensity plots in left panels. Numbers of samples for *Lbx1*: control, n = 13; *Del-R26* ^*Met*^, n = 4; for *Pax3*: control, n = 11; *Del-R26* ^*Met*^, n = 8. The sum of *Pax3* signal intensity was calculated between point A and B: A indicating a fixed position between the somites and the limb whereas B being placed at a fixed distance from A. Note almost lack of signal in *Del-R26* ^*Met*^ mutants. Scale: 500μm. Mann-Whitney and Student-*t* test.

Intriguingly, the limb phenotype of the *Del-R26* ^*Met*^ mice is reminiscent of the phenotype we reported in *Met* ^*grb2/grb2*^, *Met* ^*2P/2P*^, and *Met* ^*2S/2S*^ specificity-switch signalling mutants [18, 23, 28]. In these *Met* signalling mutants, Met-dependent migration of myoblasts is severely compromised, leading to a reduction of limb muscles more pronounced in the dorsal than in the ventral limb compartment [18, 23, 28]. We therefore assessed whether the development of migrating myoblasts was also compromised in *Del-R26* ^*Met*^ mice. We addressed this issue using distinct markers of the myogenic program [29]. Whole mount ISH on E10.5 embryos using *Lbx1* and *Pax3* probes revealed a drastic reduction of migrating myoblasts from the somites towards the limbs, with only few dispersed migrating cells left in *Del-R26* ^*Met*^ embryos compared to controls (Fig 2A and 2B). Quantification of the ISH signal throughout the limbs revealed approximately 90% reduction of migrating myoblasts in *Del-R26* ^*Met*^ forelimbs compared to controls (Fig 2C and 2D). Impaired migration of myoblasts towards the tongue was also found in *Del-R26* ^*Met*^ embryos (Fig 2A and 2B), indicating that ubiquitous expression of the Met^tg^ also compromises the developmental program of these migrating myoblasts. Together, these findings show that ubiquitously enhancing wild-type Met-RTK specifically interferes with the limb muscle developmental program by perturbing myoblast migration.

### Enhanced wild-type Met expression is permissive in migrating myoblasts, but not in limb mesenchyme

**Fig 3:**
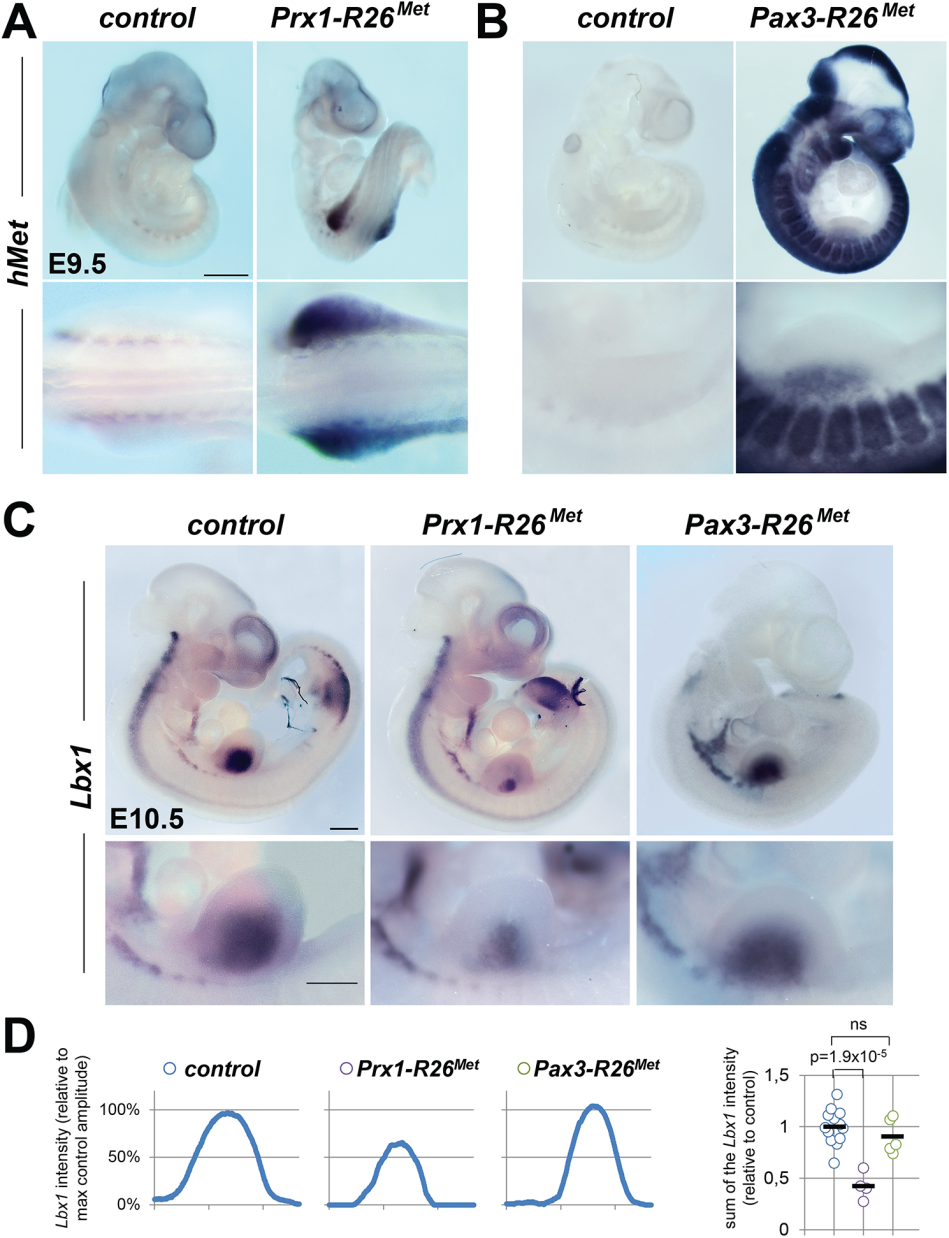
Ectopic Met in limb mesenchyme drastically reduces limb colonization by migrating myoblasts. (A, B) Whole mount ISH using the human *Met* probe showing the domain with Cre recombinase activity in *Prx1-R26* ^*Met*^ (A) and *Pax3-R26* ^*Met*^ (B) embryos. Note that: a) in *Prx1-R26* ^*Met*^ embryos, the expression of the *Met* ^*tg*^ (detected by *hMet* probe) is restricted in forelimbs; b) in *Pax3-R26* ^*Met*^ embryos, *Met* ^*tg*^ expression is found in *Pax3*-positive territories. (C) Whole mount ISH of E10.5 embryos with the *Lbx1* probe showing drastic reduction of migrating myoblasts in the forelimbs of *Prx1-R26* ^*Met*^, but not of *Pax3-R26* ^*Met*^ mutants. Note that intact migration of myoblasts towards the forming tongue (arrowhead) in *Prx1-R26* ^*Met*^ mutants correlates with the restricted expression of enhanced Met in limb mesenchyme. Bottom panel reports an enlargement at forelimb levels. (D) Quantification analyses of *Lbx1* positive domain in forelimbs. Left panels: each plot represents the average signal distribution along the white line in forelimbs. Right panel: quantifications and statistical analyses of the sum of signal intensity based on intensity plots in left panels. Numbers of samples for *Lbx1*: control, n = 13; *Prx1-R26* ^*Met*^, n = 4; *Pax3-R26* ^*Met*^, n = 5. Note the reduced *Lbx1* level in *Prx1-R26* ^*Met*^ mutants. Mann-Whitney and Student-*t* test.

The intriguing similarity of the limb muscle phenotype in gain-of-function *Del-R26* ^*Met*^ and in loss-of-function *Met* specificity-switch signalling mutants [18, 23, 28] could be interpreted in two ways. One scenario could be that in myoblasts, the amount of Met signalling must be qualitatively and/or quantitatively maintained within a narrow range. If the signalling level is reduced (as in *Met* specificity-switch signalling mutants) or enhanced (as in *Del-R26* ^*Met*^ mutants), the migration program may not occur. Alternatively, exogenous Met expression might act in limb mesenchymal cells by altering their permissiveness to invading myoblasts. To discriminate between these two possibilities, we selectively enhanced Met expression in either migrating myoblasts or in limb mesenchyme, using the *Pax3-Cre* knock-in or the *Prx1-Cre* transgenic lines, respectively [30, 31]. Consistently, whole mount ISH in *Prx1-R26* ^*Met*^ and *Pax3-R26* ^*Met*^ mutants confirmed that expression of the *Met* ^*tg*^ is adequately targeted to *Prx1* and *Pax3* expression domains, respectively (Fig 3A and 3B). Whole mount ISH with *Lbx1* and *Pax3* probes unequivocally revealed a significant impairment in myoblast migration in *Prx1-R26* ^*Met*^, but not in *Pax3-R26* ^*Met*^ mutants (Figs 3C, S5A and S5B). Quantification analyses revealed approximately 50% reduction of limb myoblasts in *Prx1-R26* ^*Met*^ mutants compared to *Pax3-R26* ^*Met*^ and control embryos (Figs 3D, S5C and S5D). At E12.5, limb-forming muscles were reduced in *Prx1-R26* ^*Met*^, but not in *Pax3-R26* ^*Met*^ embryos, as revealed by *MyoD* ISH (S5E Fig), even though the severity of this phenotype appeared less pronounced compared to earlier stages. Finally, *Prx1-R26* ^*Met*^ mice showed hyperflexed forelimbs and weak hindlimbs at birth (S5E Fig; 90%, n = 16), whereas *Pax3-R26* ^*Met*^ mutants were indistinguishable from controls. Together, these results disqualify myoblasts and identify limb mesenchymal cells as the cell type in which enhanced Met signalling acts to disrupt myoblast migration. This suggests that myoblast migration towards limb mesenchyme is impaired when exogenous Met expression overlaps with the endogenous source of HGF.

The myoblast migration defect in *Prx1-R26* ^*Met*^ mutants underlines the importance of spatial restriction of Met expression during development. We therefore asked whether alteration of the regionalised distribution of Met also interferes with the molecular program regulating limb patterning and skeletal morphogenesis. We followed expression of *Shh* and *Fgf8*, two major regulators of early limb patterning and growth, and found no obvious defects in *Del-R26* ^*Met*^ mutants compared to controls at E10.5 (S6A Fig). Furthermore, skeletal staining analyses did not reveal any major defects in bone and cartilage formation in *Del-R26* ^*Met*^ forelimbs at birth (S6B Fig). Together, these findings highlight the robustness of limb skeletal patterning program, ensured by the intercalation of multiple signalling components, which does not permit interference by ectopic Met.

**Fig 4:**
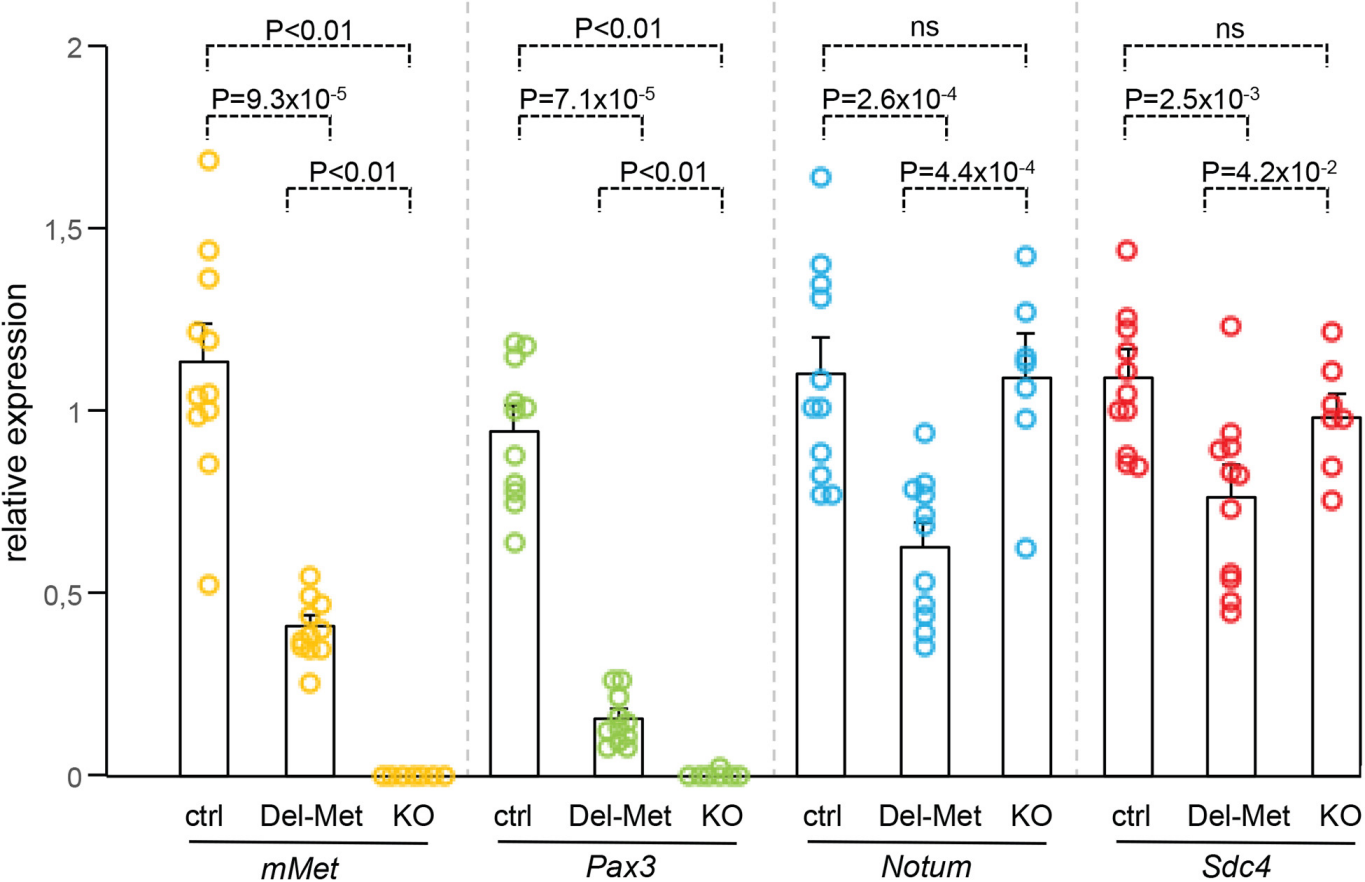
Ectopic Met in limb mesenchyme down-regulates the expression levels of *Notum* and *Syndecan4*. qRT-PCR analysis of transcript levels of mouse *Met* (*mMet*), *Pax3*, *Notum*, and *Syndecan4* (*Sdc4*) in controls (ctrl; n = 11), *Del-R26* ^*Met*^ (Del-Met; n = 11), *Met* ^*LacZ/d(neo)*^ (KO; n = 7). Each dots corresponds to transcript levels in forelimbs of E10.5 individual embryos (done in triplicate). Columns correspond to the average value, expressed as mean ± s.e.m. Note: downregulation of *mMet* and *Pax3* in *Del-R26* ^*Met*^ and *Met* ^*LacZ/d(neo)*^ mutants compared to control, consistent with lack of migrating myoblasts; downregulation of *Notum* and *Syndecan4* in *Del-R26* ^*Met*^ mutants compared to control, whereas no significant changes were found in *Met* ^*LacZ/d(neo)*^ mutants. Mann-Whitney and Student-*t* test.

We next analysed whether alteration of the regionalised distribution of Met leads to molecular changes in limb buds by following mainly the expression levels of cell surface proteins such as heparan-sulfate proteoglycans (HSPGs), glypicans [32–34] and syndecans [35], which act as gatekeepers of cellular responses by modulating extracellular signal distribution and perception by targeted cells. We also analysed the expression levels of HSPG modifiers, such as *Notum*, *Hst2st1*, and *Hst3st1* [36–38]. Among these candidates, some have been previously reported to also modulate HGF signalling [39–41] or to be regulated by HGF/Met [42–44]. We also analysed *Sdf1* levels for its cooperative function with Met signalling to control myoblast migration [45]. A total of 12 genes were screened: the 6 *Glypican*s, *Syndecan3* and *4*, *Hst2st1*, *Hst3st1*, *Notum*, and *Sdf1*. To analyse the expression levels of these genes, E10.5 forelimbs dissected from *Del-R26* ^*Met*^ mutants and controls were used for qRT-PCR. Studies were restricted to embryos that showed low levels of β-galactosidase activity in the whole body as a read-out of efficient activation of the Met^tg^ (see S1 Fig). We first assessed the sensitivity of this approach in detecting molecular changes possibly occurring in a limited number of cells within the limb. In particular, we quantified transcript levels of *Pax3* and mouse *Met*, both expressed in migrating myoblasts, and consistently found a drastic reduction in their mRNA levels in *Del-R26* ^*Met*^ limbs compared to controls (Fig 4). Second, we confirmed a switch in *LacZ* versus *Met* ^*tg*^ expression in control and *Del-R26* ^*Met*^ limbs (S7A Fig). We next screened the 12 selected candidates by comparing their expression levels in control versus *Del-R26* ^*Met*^ limbs. No significant changes were observed in the expression levels of all *Glypicans*, *of Syndecan3*, *Hst2st1*, *Hst3st1*, and *Sdf1* (S7B and S7C Fig). In contrast, we found that expression levels of *Notum* and *Syndecan4* were significantly reduced in *Del-R26* ^*Met*^ versus control limbs (Fig 4). We next asked whether such molecular differences in *Del-R26* ^*Met*^ mutants resulted from the depletion of limb myoblasts, or reflected expression changes in limb mesenchyme. To address this question, we took advantage of *Met* loss-of-function mutants (*Met* ^*LacZ/d(neo)*^), in which limbs are also devoid of migrating myoblasts (S8 Fig), but where no specific changes in limb mesenchyme can be expected [18, 22]. Consistently, *Pax3* and mouse *Met* myoblast-specific transcripts were also absent from *Met* ^*LacZ/d(neo)*^ mutant limbs (Fig 4). In contrast, the expression levels of *Notum* and *Syndecan4* (changed in *Del-R26* ^*Met*^ limbs) were similar in *Met* ^*LacZ/d(neo)*^ and control limbs (Fig 4). Altogether, these results exclude that downregulation of *Notum* and *Syndecan4* in *Del-R26* ^*Met*^ limbs is a consequence of lack of migrating myoblasts, and identify them as molecular changes caused by Met expression in limb mesenchyme.

### Met-expressing myoblasts are capable of buffering enhanced wild-type Met levels

**Fig 5:**
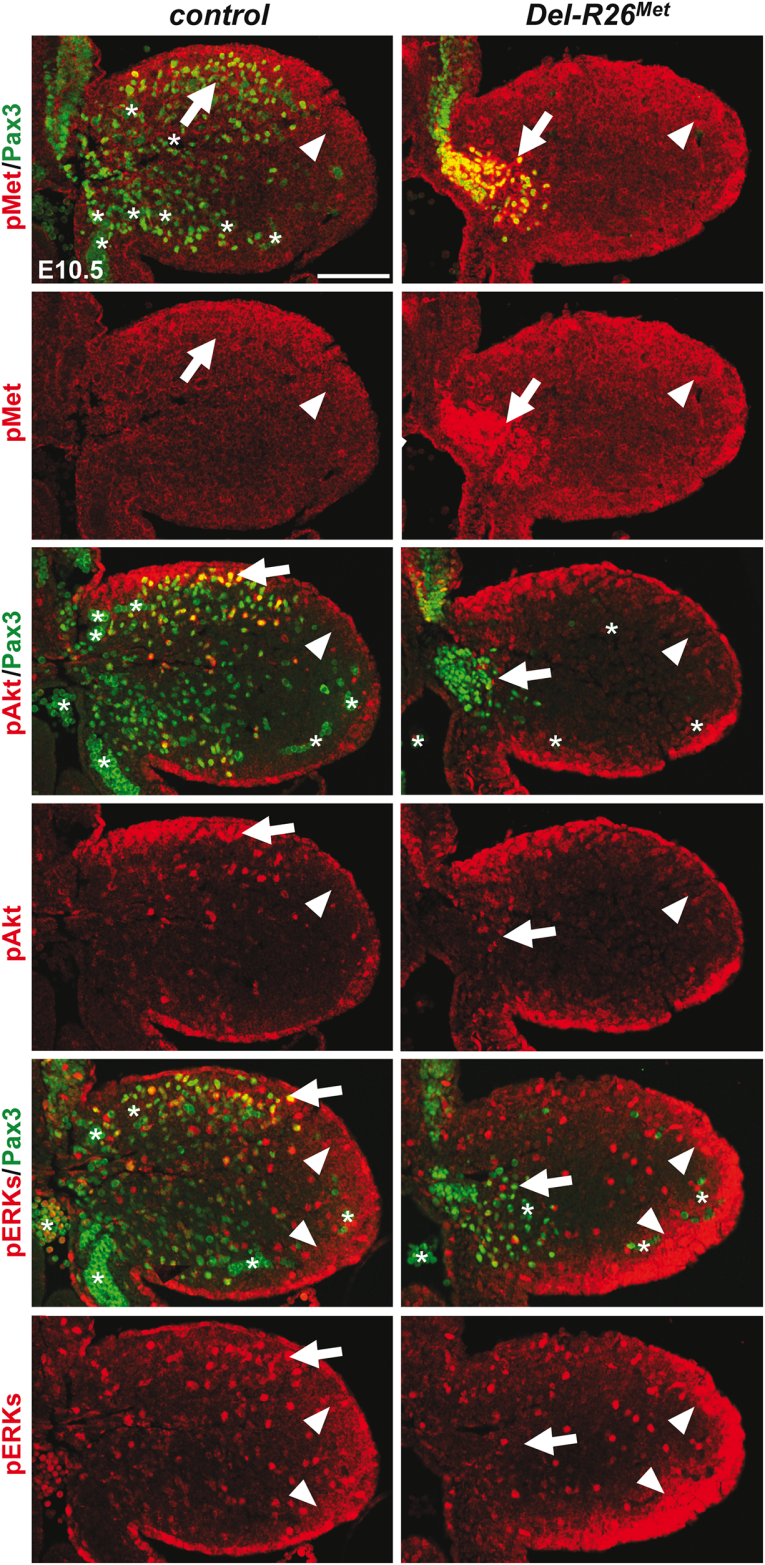
Enhanced Met expression levels in *Del-R26* ^*Met*^ myoblasts does not perturb activation of downstream signalling effectors. Limb transverse sections of E10.5 control and *Del-R26* ^*Met*^ embryos showing the distribution of phospho-Met (on Tyr_1234–1235_), phospho-Akt, phospho-ERKs (red) and of Pax3 protein (green) in myoblasts. Note ectopic phospho-Met in limb mesenchyme (arrowheads) and in non-migrating myoblasts (arrows) in *Del-R26* ^*Met*^ mutants. Asterisks indicate non-specific staining in blood cells. Scale: 100μm.

**Fig 6:**
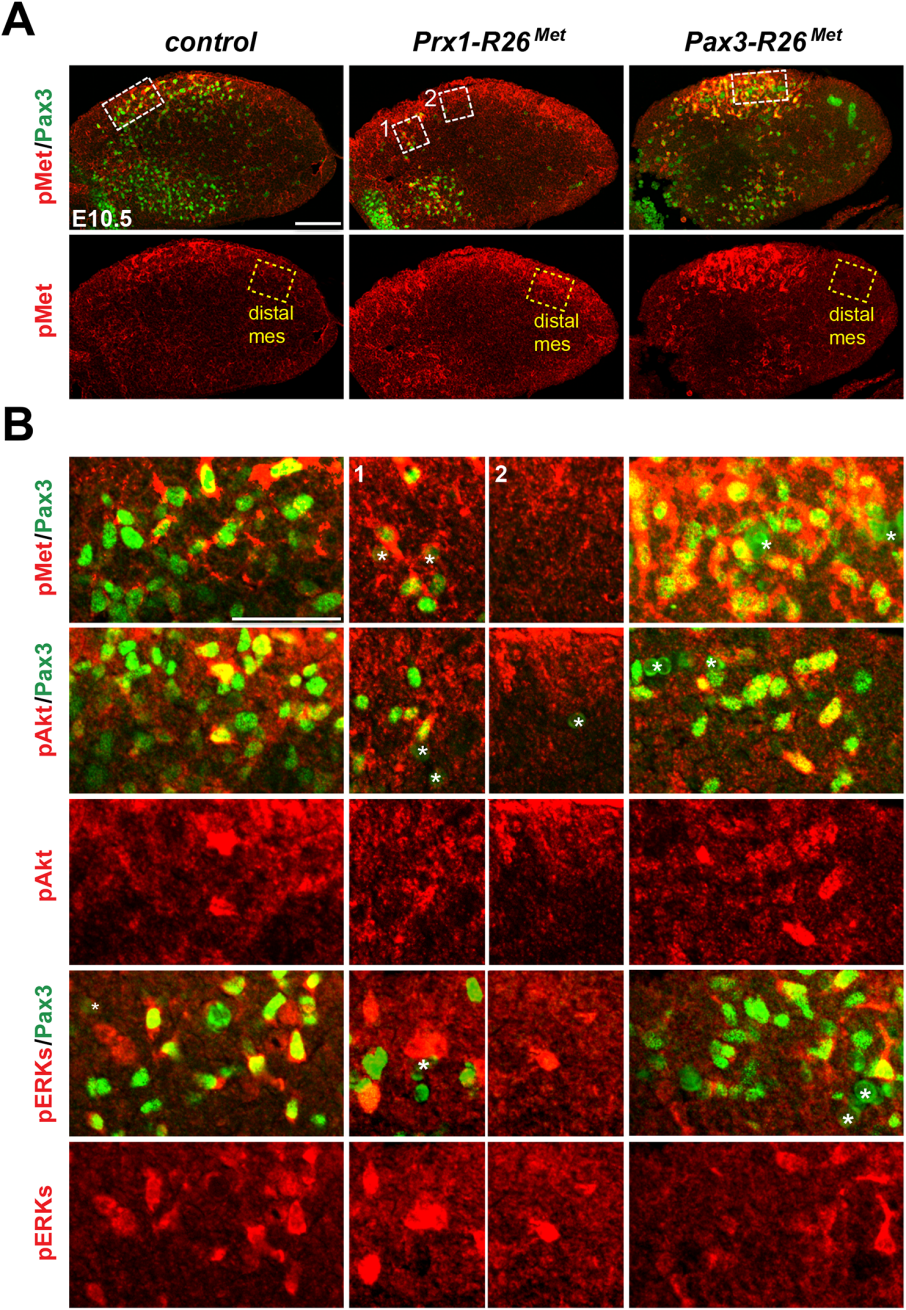
Immunohistochemical analysis showing that myoblasts possess a buffering competence to enhanced Met levels. (A) Limb transverse sections of E10.5 control, *Prx1-R26* ^*Met*^, and *Pax3-R26* ^*Met*^ embryos showing the phospho-Met (red) and Pax3-positive myoblasts (green). Note ectopic phospho-Met in limb mesenchyme of *Prx1-R26* ^*Met*^ and in migrating myoblasts of *Pax3-R26* ^*Met*^ mutants. Few migrating myoblasts are present in limb mesenchyme of *Prx1-R26* ^*Met*^ mutants. White boxes indicate the position of enlargements shown in panel B. Yellow boxes indicate distal mesenchyme (distal mes) area devoid of myoblasts where enhanced phospho-Met signal is detected in *Prx1-R26* ^*Met*^ mutants. (B) High magnification of limb transverse sections showing phosphorylation levels of Met, Akt, and ERKs (red) in migrating myoblasts (Pax3-positive; green) of E10.5 control, *Prx1-R26* ^*Met*^, and *Pax3-R26* ^*Met*^ embryos. Note that despite the high phospho-Met levels in *Pax3-R26* ^*Met*^ mutants, no major changes are observed in phospho-Akt and phospho-ERKs when compared to controls and *Prx1-R26* ^*Met*^ mutants. For *Prx1-R26* ^*Met*^ mutants, two areas are shown: square 1 indicates few migrating myoblasts; square 2 indicates mesenchyme devoid of myoblasts. Asterisks indicate non-specific staining in blood cells. Scale: 100μm.

The intact myoblast migration in *Pax3-R26* ^*Met*^ mutants could reflect either the robustness of the migration response to increased activation levels of Met intracellular effectors or an intrinsic capability of cells to restrain Met-RTK input. By performing IHC on embryo sections, we followed Met signalling levels in control and mutant forelimbs at E10.5, when myoblast migration occurs. In myoblasts, levels of pTyr_1234–1235_-Met were enhanced in *Del-R26* ^*Met*^ (non-migrating cells blocked at the base of the limb) and *Pax3-R26* ^*Met*^ (migrating cells progressing within the limb) compared to controls, whereas no major changes were observed in *Prx1-R26* ^*Met*^ (where non-migrating myoblasts are also blocked in a proximal position in the limb) (Figs 5 and 6A). By exploring the activation levels of two Met effectors, we found that the phosphorylation levels of Akt and ERKs were comparable in control, *Del-R26* ^*Met*^, *Pax3-R26* ^*Met*^, and *Prx1-R26* ^*Met*^ myoblasts, despite high levels of phospho-Met in *Del-R26* ^*Met*^ and *Pax3-R26* ^*Met*^ (arrows in Fig 5 and Fig 6B) Together, these results show that myoblasts have the competence to buffer enhanced Met levels. In contrast, in limb mesenchymal cells levels of phospho-Met were slightly increased in *Del-R26* ^*Met*^ and *Prx1-R26* ^*Met*^ when compared to control and *Pax3-R26* ^*Met*^ embryos (Figs 5 and 6A). This is best seen by comparing levels in areas devoid of myoblasts even in control embryos (arrowheads in Fig 5 and “distal mes” boxes in Fig 6A). This was accompanied by an increase in the phosphorylation levels of Akt and more moderately of ERKs (Figs 5 and 6B). These findings indicate that limb mesenchymal cells lacks the capacity to buffer an ectopic Met activation, which in turn causes defects in myoblast migration.

### Restriction of enhanced Met signalling occurs at distinct levels as revealed by biochemical studies in embryonic hepatocytes

**Fig 7:**
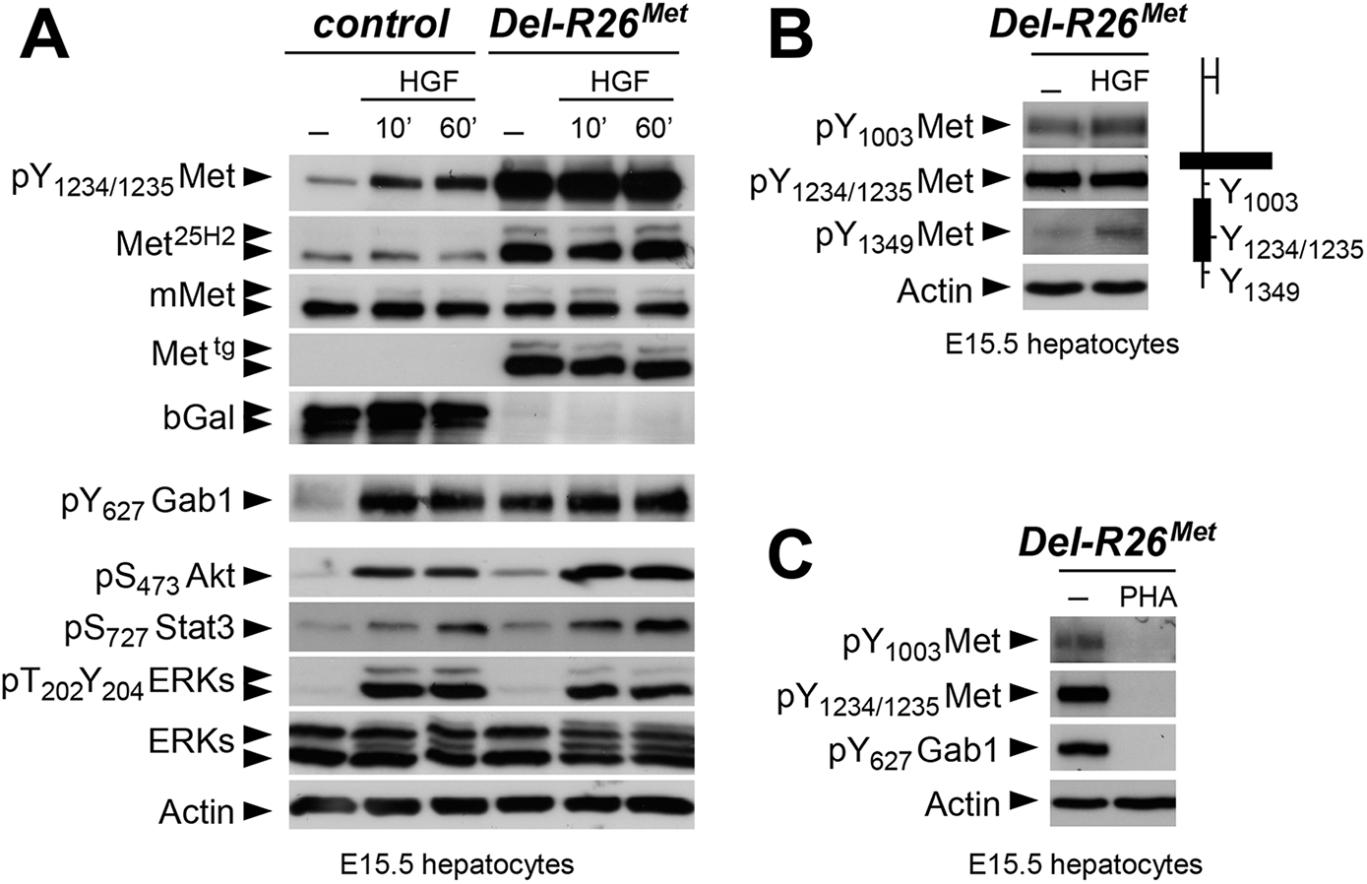
Biochemical analyses in embryonic hepatocytes show restriction of enhanced Met signalling at distinct levels. (A) Western blot analyses of total protein extracts of E15.5 primary embryonic hepatocytes derived from *R26* ^*stopMet*^ (containing the LacZ-stop cassette therefore expressing the β-galactosidase) and from *Del-R26* ^*Met*^ (after deletion of the LacZ-stop cassette, therefore expressing the transgenic Met detected by anti-human Met antibodies) embryos. Analyses was performed before and after HGF stimulation (50ng/ml). Note: a) similar levels of mouse Met in *R26* ^*stopMet*^ and *Del-R26* ^*Met*^ cells; b) high levels of Met phosphorylation on Tyr_1234–1235_ in *Del-R26* ^*Met*^ cells independently of HGF stimulation; c) comparable Gab1, Akt, Stat3, and ERK phosphorylation levels upon HGF stimulation in *R26* ^*stopMet*^ and *Del-R26* ^*Met*^ cells; d) despite Gab1 phosphorylation in untreated *Del-R26* ^*Met*^ cells in contrast to control *R26* ^*stopMet*^ cells, the phosphorylation levels of Akt, Stat3, and ERK is unchanged. Actin protein levels were used as loading controls in all western blot analyses. (B) Left: western blot analyses of total protein extracts of E15.5 primary embryonic hepatocytes derived from *Del-R26* ^*Met*^ embryos. Note basal levels of Met phosphorylation on Tyr_1003_ and on Tyr_1349_; phosphorylation levels are further increase upon HGF stimulation (50ng/ml). Right: schematic representation of Met indicating the different tyrosine residues analysed by western blots. (C) Western blot analysis of total protein extracts of E15.5 primary embryonic hepatocytes derived from *Del-R26* ^*Met*^ embryos showing impaired phosphorylation of Met and Gab1 in the presence of the Met inhibitor PHA665752 (PHA; 1μM).

We next explored how buffering of enhanced Met signalling occurs in cells. Because of technical difficulties in isolating and establishing primary myoblast cultures from E10.5 embryos for quantitative western blot studies, we addressed this issue using primary embryonic hepatocytes in which endogenous Met is expressed and required for their survival, as shown through our earlier studies of *Met* signalling mutants [18, 28, 46–48]. Primary embryonic hepatocyte cultures were established from E15.5 *R26* ^*stopMet*^ and *Del-R26* ^*Met*^ embryos and used to follow biochemical changes in the Met signalling cascade. As expected, *Del-R26* ^*Met*^ embryonic hepatocytes express the Met^tg^, but not the β-galactosidase (in the stop cassette; Fig 7A). Total Met protein levels in *R26* ^*stopMet*^ and *Del-R26* ^*Met*^ primary embryonic hepatocytes were assessed by using the Met^25H2^ antibodies generated against a synthetic peptide containing the amino acids surrounding the Tyr_1234–1235_ within the kinase domain (recognising human and mouse Met). Quantification of the Met^25H2^ levels revealed an approximately 4-fold increase of total Met protein amount in *Del-R26* ^*Met*^ versus *R26* ^*stopMet*^ controls. Increased levels of Met proteins correlate with a concomitant increase in Met phosphorylation levels on Tyr_1234–1235_ (critical for Met kinase activation; visualized with an antibody recognising mouse and human phosphorylated Met), which are not further enhanced upon HGF stimulation (Fig 7A). We found two intriguing aspects of enhanced Met expression on downstream components of the Met signalling cascade. Concerning Gab1, a cytoplasmic protein directly recruited by Met and functioning as a platform for Met effectors, we found that: a) it was already phosphorylated in *Del-R26* ^*Met*^ cells to a level comparable to that of control cells upon HGF stimulation, b) its phosphorylation levels in *Del-R26* ^*Met*^ cells was not further enhanced upon HGF stimulation (Fig 7A). Concerning further downstream Met effectors such as Akt, Stat3, and ERKs, we found that their phosphorylation levels increased upon HGF stimulation with comparable kinetic profiles in *Del-R26* ^*Met*^ and control cells (Fig 7A). These findings indicate that the constitutive enhancement of Met expression and phosphorylation on Tyr_1234–1235_ in hepatocytes does not result in a constitutive activation of downstream signalling components.

To get insights into how enhanced Met signalling is restricted in embryonic cells, we analysed the phosphorylation levels of two other tyrosine residues implicated in receptor signalling and endocytosis. Intriguingly, we found basal levels of Met phosphorylation on Tyr_1003_ (critical for Met protein ubiquitination and degradation) and on Tyr_1349_ (one of the two multifunctional docking sites required for Met signalling), which were enhanced upon HGF stimulation (Fig 7B). Consistently, Met and Gab1 phosphorylation was impaired in the presence of the Met inhibitor PHA665752 (Fig 7C). Thus, basal levels of Met phosphorylation in mutant embryonic hepatocytes appear sufficient to initiate signalling activation (e.g. Gab1 phosphorylation on Tyr_627_), but not to propagate signalling activation to downstream pathways (e.g. Akt, Stat3, ERKs). Together, these findings indicate that the Met^tg^ achieves its full signalling competence following a burst of HGF stimulation.

### Gain of wild-type Met expression in limb mesenchyme does not perturb expression pattern and levels of its ligand HGF, but its bioavailability

**Fig 8:**
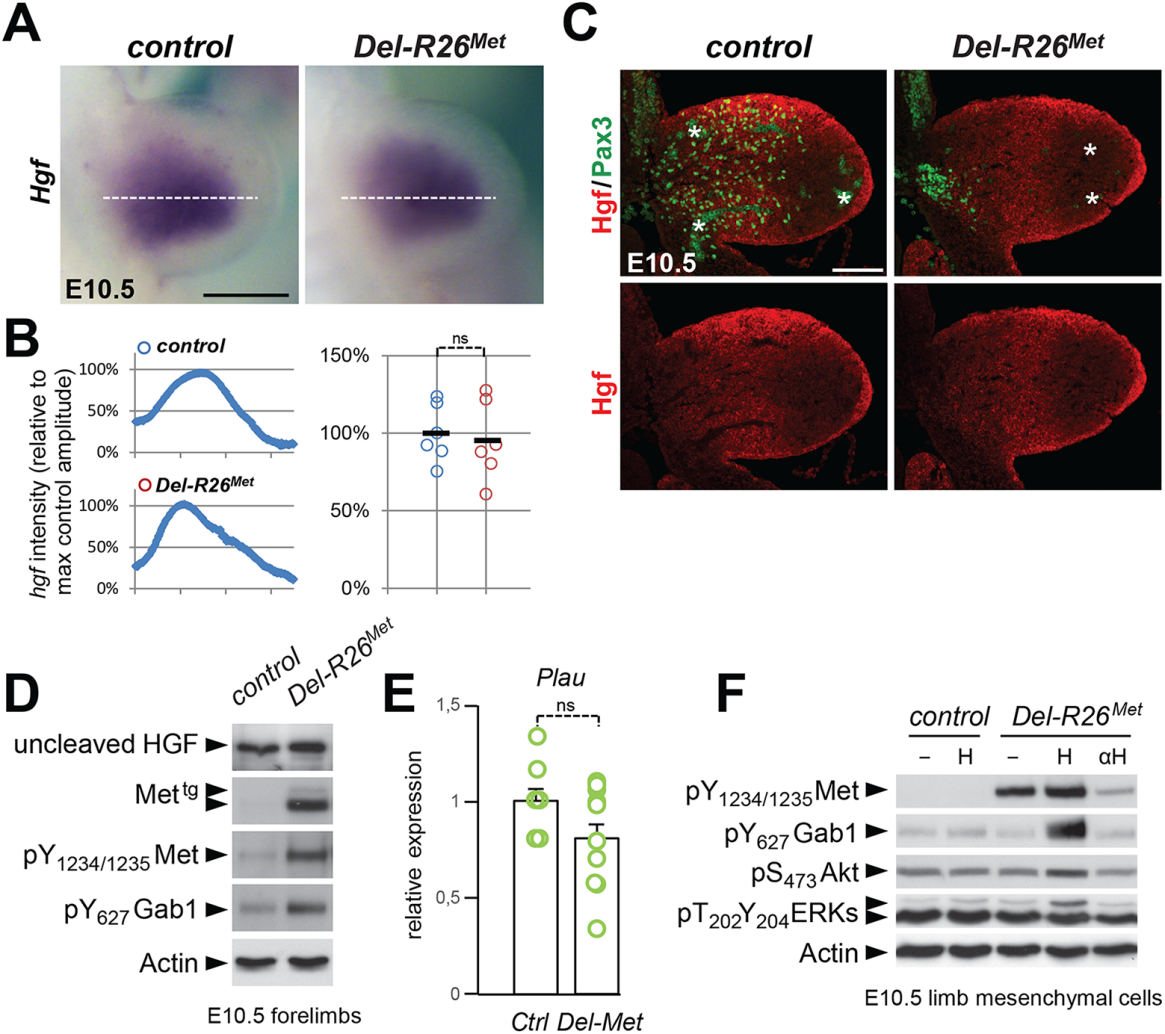
Ectopic Met does not change the expression profile and levels of HGF in limb mesenchyme. (A) Whole mount ISH of E10.5 control and *Del-R26* ^*Met*^ embryos with *Hgf* probe showing comparable expression domain in developing forelimbs. (B) Quantification analyses of *Hgf* positive domains in forelimbs. Left panels: each plot represents the average signal distribution along the white line in forelimbs. Right panels: quantifications and statistical analyses of the sum of signal intensity based on intensity plots in left panels. Numbers of samples: control, n = 6; *Del-R26* ^*Met*^, n = 6. Note no significant changes of signal in controls and *Del-R26* ^*Met*^ mutants. (C) Immunofluorescence analysis of HGF (red) and Pax3 (green) proteins in transversal sections of E10.5 control and *Del-R26* ^*Met*^ embryos at forelimb levels. Note the impaired myoblast migration (Pax3-positive cells) in *Del-R26* ^*Met*^ mutants, whereas no significant changes are detected in HGF protein levels. Asterisks indicate non-specific staining in blood cells. Scale: 100μm. (D) Western blot analyses of total protein extracts of E10.5 control and *Del-R26* ^*Met*^ dissected forelimbs. Note comparable levels of uncleaved HGF in *Del-R26* ^*Met*^ mutants despite the ectopic expression of Met^tg^ and the enhanced phosphorylation levels of Met and Gab1. (E) qRT-PCR analysis showing comparable *Plau* transcript levels in E10.5 dissected forelimbs of control (ctrl; n = 11) and *Del-R26* ^*Met*^ (Del-Met; n = 11) embryos. (F) Western blot analysis of total protein extracts of E10.5 forelimb mesenchymal cell cultures showing phosphorylation levels of Met, Gab1, Akt, and ERKs in control and *Del-R26* ^*Met*^ cultures, in cells untreated, stimulated with HGF (H) or in the presence of anti-HGF blocking antibodies (αH).

As the domain of Prx1-cre activity in the limb encompasses the source of HGF exerting chemoattraction on migrating myoblasts, we next asked whether ectopic Met in these cells alters the bioavailability of HGF to migrating cells. ISH and IHC analyses revealed no significant differences on HGF expression patterns or levels between *Del-R26* ^*Met*^ and control embryos (Fig 8A, 8B and 8C), ruling out the possibility that ectopic Met alters *Hgf* gene expression and HGF protein production in limb mesenchyme. Moreover, we found comparable protein levels of uncleaved HGF in E10.5 *Del-R26* ^*Met*^ and control forelimbs (Fig 8D) as well as unchanged mRNA levels of urokinase-type plasminogen activator (*Plau*), an enzyme involved in HGF processing (Fig 8E).

Next, we investigated whether Met expression in mesenchymal territories would alter HGF bioavailability. For this purpose, we established primary embryonic mesenchymal cell cultures from E10.5 forelimbs and biochemically assessed their competence to respond to HGF stimulation. As expected, control limb mesenchymal cells did not respond to HGF stimulation, consistent with the fact that these cells do not express the receptor (Fig 8F). In contrast, we found high levels of Met phosphorylation on Tyr_1234–1235_ in *Del-R26* ^*Met*^ limb mesenchymal cells (Fig 8F). HGF stimulation induced Gab1 phosphorylation and enhanced the basal levels of Akt and ERKs phosphorylation (Fig 8F). Furthermore, treatment with anti-HGF blocking antibodies drastically reduced the phosphorylation levels of Met in *Del-R26* ^*Met*^ limb mesenchymal cells, thus showing that HGF is indeed produced by these cells and that basal Met phosphorylation is due to endogenous HGF in these culture conditions (Fig 8F). Finally, we biochemically analysed the levels of HGF in conditioned media of *Del-R26* ^*Met*^ limb mesenchymal cells and found that levels of uncleaved HGF were comparable to those of control cells (S9A Fig).

**Fig 9:**
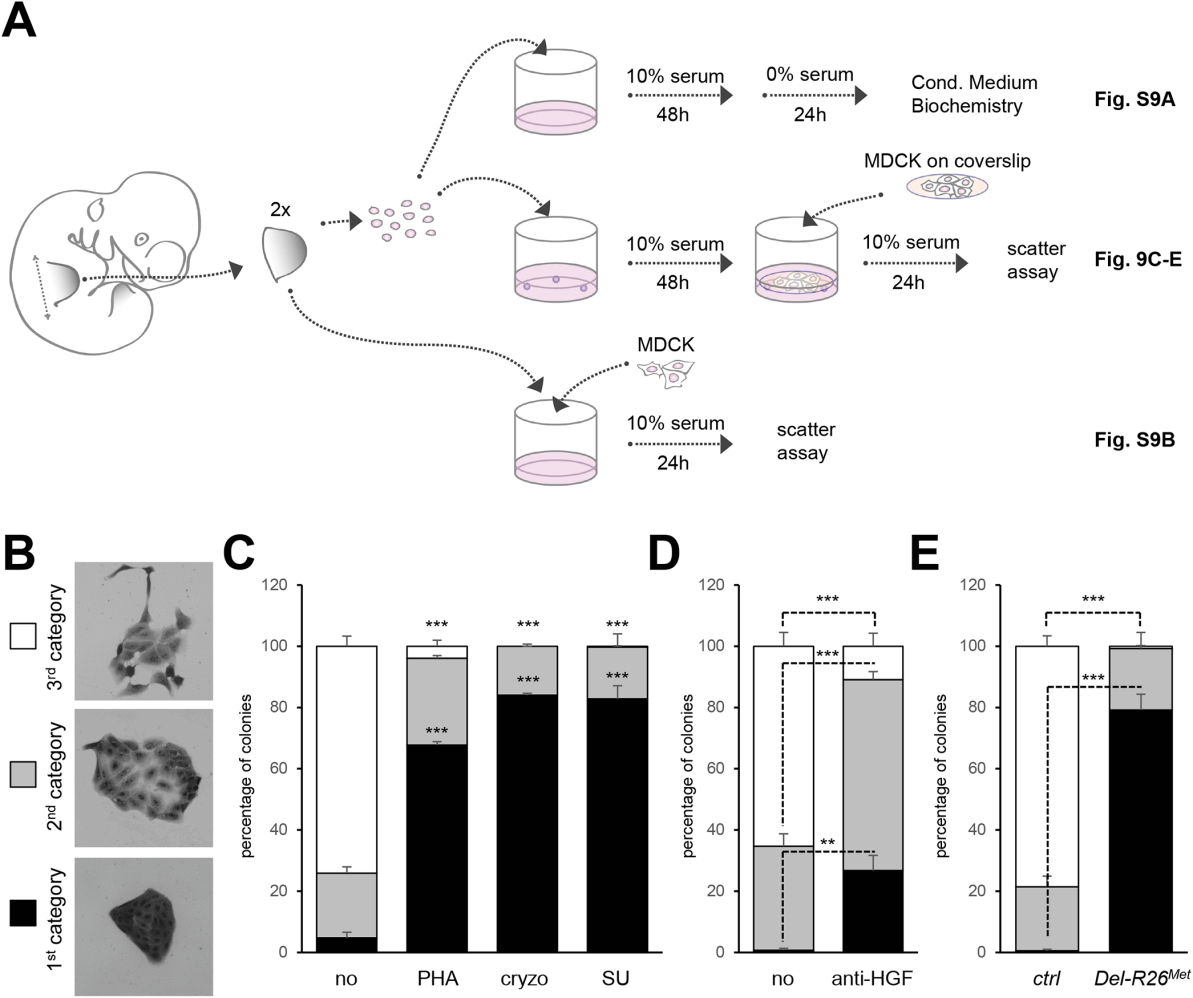
Ectopic Met in limb mesenchyme alters HGF bioavailability. (A) Scheme illustrating the experimental procedure employed for evaluating through MDCK cell scattering the bioavailability of HGF from control and *Del-R26* ^*Met*^ mutant limb mesenchymal cells or from dissected forelimbs. The scheme indicates the experimental procedure applied for collecting media conditioned by limb mesenchymal cells for biochemical analysis (top; shown in S9A Fig), for MDCK scattering assays using co-cultures with limb mesenchymal cells (middle; shown in Fig 9C, 9D and 9E) or with dissected limbs (bottom; shown in S9B Fig). (B) Pictures of MDCK colonies showing the three categories that were defined to determine the extent of cell contact and spreading for quantification studies of scattering response. (C) Quantitative analysis of MDCK cell scattering in co-cultures with control limb mesenchymal cells in the absence (no) and in the presence of the Met inhibitor PHA665752 (PHA; 1μM), cryzotinib (Cryzo; 1μM), or SU11274 (SU; 1μM). (D) Quantitative analysis of MDCK cell scattering in co-cultures with control limb mesenchymal cells in the absence (no) and in the presence of the anti-HGF blocking antibodies (anti-HGF; 30μg/ml). (E) Quantitative analysis of MDCK cell scattering in co-cultures with control or *Del-R26* ^*Met*^ limb mesenchymal cells. Note a drastic reduction in the scattering response when MDCK cells are co-cultured with *Del-R26* ^*Met*^ mutant cells (control: n = 4; *Del-R26* ^*Met*^: n = 3). Mann-Whitney and Student-*t* test.

To explore whether the HGF released by control and *Del-R26* ^*Met*^ cultures was bioactive, we co-cultured MDCK cells with E10.5 dissociated limb mesenchymal cells as a source of HGF (Fig 9A). MDCK cells acquire a “scattered phenotype” upon HGF stimulation [49, 50], thus providing an excellent readout of HGF bioavailability in conditioned media by limb mesenchymal cells. To evaluate HGF bioavailability, we established an experimental setting in which MDCK cells were co-cultured either with dissociated limb mesenchymal cells or with dissected limbs (Fig 9A). HGF activity was quantified by scoring the percentage of MDCK clones exhibiting a dense phenotype (category I), an expanded phenotype (category II), or a scattered phenotype with some or several cells detached from the clone (category III; Fig 9B). A condition with mesenchymal cells from two control forelimbs consistently triggered an efficient MDCK scattering response (Fig 9C). MDCK scattering response triggered by conditioned media from control limb mesenchymal cells was drastically reduced in the presence of Met inhibitors (PHA665752, cryzotinib, or SU11274: 1μM; Fig 9C) or of anti-HGF blocking antibodies (anti-HGF: 30μg/ml; Fig 9D). In contrast, we found a drastic reduction in the percentage of MDCK colonies with a scattered phenotype using mesenchymal cells from two *Del-R26* ^*Met*^ forelimbs (Fig 9E). Similar results were obtained using MDCK cells directly co-cultured with E10.5 forelimb explants (S9B Fig). Together, these findings indicate that although ectopic Met expression in limb mesenchyme does not alter HGF expression pattern or levels, it impairs bioavailability of HGF from mesenchymal cells.

## Discussion

**Fig 10:**
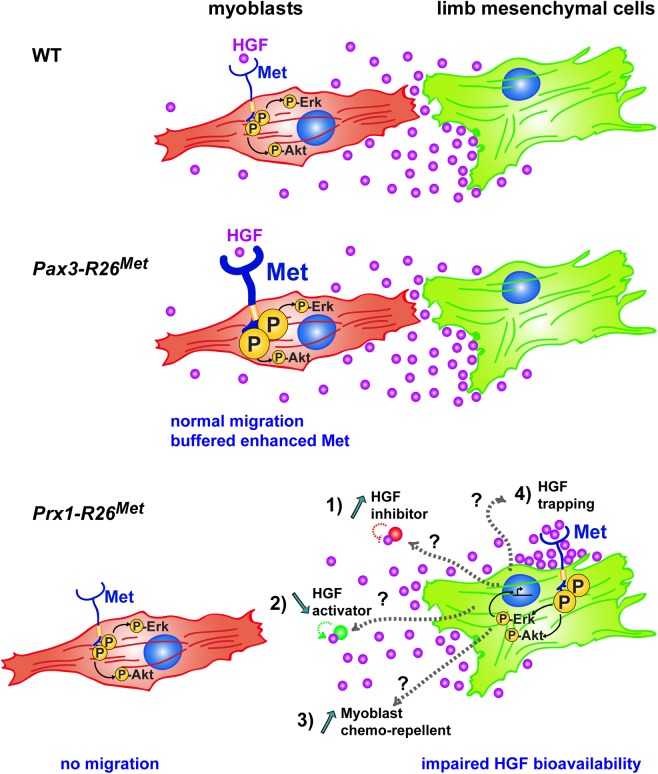
Schematic representation summarizing the different molecular and phenotypic effects of enhanced Met expression in myoblasts and limb mesenchymal cells. In a wild-type context (top), limb mesenchymal cells secrete HGF required for migration of myoblasts towards the limb buds. Enhanced expression of Met in myoblasts (as assessed in *Pax3-R26* ^*Met*^ embryos; middle) does not alter their migration due to a buffering event: the activation levels of signalling effectors such as ERKs and Akt are restrained despite enhanced Met phosphorylation. The size of each signal is representative of their phosphorylation levels. Limb mesenchymal cells are vulnerable to ectopic Met expression (as assessed in *Prx1-R26* ^*Met*^ embryos; bottom), illustrated by changes in gene expression, by failure of HGF bioavailability, and by myoblast migration defects. Alteration of HGF bioavailability can be due to: 1) upregulation of a negative interactor that would interfere with the capacity of HGF to bind/activate Met (indicated as “HGF inhibitor”), 2) downregulation of a HGF interactor acting as enhancer of its bioactivity (indicated as “HGF activator”), 3) expression of a chemorepellent factor that renders limb mesenchyme inaccessible to migrating myoblasts, or 4) HGF titration by ectopic Met in mesenchymal cells (indicated as “HGF trapping”).

The events that cells experience overtime shape progressively their intrinsic capability to respond and to integrate RTK inputs. Our data illustrate that enhanced wild-type RTK signalling is differentially interpreted by cells according to their competence and the robustness of developmental programs they undergo. Most developing cell types appear insensitive to changes in Met expression either because enhanced Met protein levels is not followed by its activation (e.g. in the absence of ligand), or because it is neutralized by the action of other signals acting on cells. This is not the case of migrating myoblasts that, although insensitive themselves to enhanced Met expression, failed to migrate towards the limb buds as a result of ectopic Met expression in mesenchymal cells. Ectopic Met in the mesenchyme perturbs transcriptional regulation of some genes and interferes with HGF bioavailability, thus rendering the mesenchyme non accessible to myoblasts (Fig 10). Our results demonstrate that whereas most cell types during development are capable of handling moderate perturbations in RTK signalling levels, certain cell types are vulnerable to alterations in changes of spatial RTK action.

### Cell competence to Met activation

The strategy of using the wild-type Met-RTK, rather than constitutive active forms, to generate *Del-R26* ^*Met*^ mice has enabled expression of the receptor at more physiological levels. A moderate increase in Met levels during development drastically affects limb muscle formation by perturbing myoblast migration. Although we cannot exclude subtle phenotypes in other developing organs, these results highlight the restricted sensitivity of the myoblast migration process to ectopic Met in limbs. We genetically demonstrate that myoblasts themselves are not susceptible to enhanced Met signalling levels for their migration, when gain of expression is achieved in the myogenic lineage with *Pax3-Cre*. This context contrasts with a genetic setting in which *Pax3* gain-of-function triggers ectopic myoblast delamination and migration at non-limb somite levels, caused by Met over-expression [51]. It is therefore tempting to propose the existence of a threshold level of sensitivity to enhanced Met signalling for migrating myoblasts: Met signalling is enhanced below this level in *Del-R26* ^*Met*^, thus failing to elicit any qualitative change in myoblast behaviour, whereas Met signalling is enhanced above this threshold in *Pax3* gain-of-function mutants, thus triggering ectopic delamination. However, it is also possible that the effects of *Pax3* gain-of-function mutants involve missregulation of other *Pax3* target genes. The *Met* specificity-switch mutants have previously revealed that myoblast migration is impaired by lowering qualitative and/or quantitative Met signalling levels [18, 28]. Thus, findings from the present studies together with those discussed here highlight the existence of a precise window of Met signalling compatible with proper myoblast migration: within this window, cells can buffer fluctuations in RTK signalling input to avoid changes in qualitative output. This also implies that myoblasts are competent to read Met signalling for proper migration within a defined quantitative range.

### Modulating HGF bioavailability in limb mesenchyme for muscle patterning

Our studies also uncover another intriguing aspect linked to myoblasts migration: their susceptibility to alterations in the spatial distribution of Met expression. The limb mesenchyme is a local source of HGF exerting a chemoattractant effect on myoblasts that migrate from the somites. In a wild-type context, migrating myoblasts experience a territory devoid of Met-positive cells along their path. Our results ruled out that ectopic Met would act by altering the HGF mRNA and protein expression levels in limb mesenchyme. Instead, they provide evidence that limb mesenchyme must be devoid of Met expressing cells to ensure normal HGF bioavailability. Although we cannot fully exclude that ectopic Met acts by titrating the HGF secreted by limb mesenchyme, a series of experimental arguments make this possibility unlikely. Comparable levels of HGF protein were found in conditioned media from control and *Del-R26* ^*Met*^ limb mesenchymal cells, in spite of reduced scattering-induced activity, indicating that HGF secretion and release is not impaired by the occurrence of autocrine HGF/Met binding. Moreover, an event of HGF trapping by Met should recapitulate all developmental defects observed in *Met* loss-of-function mutants. Although the molecular mechanism remains to be identified, it is tempting to speculate that defects in HGF bioavailability could be linked to transcriptional and/or posttranscriptional changes caused by ectopic Met in limbs. This possibility is supported by our data showing signalling (e.g. phospho-Met, phospho-Gab, and phospho-ERKs upregulation) and transcriptional (e.g. *Notum* and *Syndecan4* downregulation) changes occurring in mutant limbs compared to controls. Thus, ectopic Met expression and activation in limb mesenchyme triggers a number of qualitative responses that cannot be buffered by cells. Our co-culture studies highlight a difference in HGF bioavailability, which could result from a change in expression levels of a modulator of HGF, such as either the downregulation of a HGF interactor acting as enhancer of HGF bioactivity, or the upregulation of a negative interactor that would interfere with the capacity of HGF to bind/activate Met (Fig 10). Among positive modulators, it has been shown that Glypican1 regulates HGF/Met-triggered migration of C_2_C_12_ myoblasts [52], Syndecan4 is expressed together with Met in adult satellite cells where it contributes to muscle regeneration [53, 54], and Glypican4 sustains HGF-mediated branching morphogenesis [39]. Alternatively, mutant cells could express a chemorepellent factor that would render the limb mesenchyme inaccessible to migrating myoblasts (Fig 10). The identification and the functional validation of the molecular mechanism(s) responsible for the non-cell autonomous defect of myoblast migration in *Del-R26* ^*Met*^ mutants is highly challenging, as: 1) a putative HGF modulator could be altered at the level of transcription, post-transcription, and/or secretion; 2) modulators of chemoattractants do not exert an “on or off” action, but rather fine tune signalling mechanisms, and may act redundantly with one another; 3) distinct mechanisms could cooperate to cause this qualitative phenotype, each of them participating although with a variable quantitative extent.

### Robustness versus sensitiveness of developmental programs to fluctuation on RTK signalling levels

By using motor neurons as a cellular paradigm, we have provided examples of robustness versus sensitivity to RTK signalling in developmental outcomes [22, 23]. We have shown that a specific motor neuron pool requires HGF/Met for survival, hence for proper maintenance of muscle innervation. In contrast, neighbouring Met-expressing motor neuron pools do not depend on the action of HGF/Met for their survival, in spite of their early dependence for motor neuron subtype specification and axon growth [22]. Furthermore, we have shown that whereas HGF/Met-dependent motor neuron survival and fate specification is permissive to substitutions of downstream signalling routes, nerve growth patterning is strictly dependent on a selective, non-substitutable, pathway [23]. Intriguingly, signalling requirements for cell survival in motor neurons is different than those in hepatocytes [46–48], demonstrating how signalling requirements for RTK-triggered biological outcomes is refined by the type of cells. Altogether, these results are consistent with the model whereby the degree of equivalence between signalling cascades available to RTK-mobilisation within a developmental program for a given biological outcome is refined in a cell type-dependent manner, thus determining the degree of robustness versus sensitiveness to RTK signals according to cell competence.

The present study further expands our knowledge on robustness versus sensitiveness of cells in developmental processes with respect to subtle increases in RTK signalling. Besides the sensitivity of myoblasts to changes in Met signalling in limb mesenchyme, most other developmental processes appear robust and capable of buffering excess Met-RTK signalling (Fig 10). How can most cells deal with RTK signalling perturbation? For cells with endogenous Met, one possibility is that they can buffer the additional burst of RTK signalling by “considering” it as a negligible quantity with respect to the overall signalling network operating in cells. Such buffering competence—or resilience—would be possible as long as this additional burst of RTK occurs within a defined quantitative window. Our biochemical studies show that effectiveness of the enhanced Met expression and activation is limited at different intracellular levels. A first point of restriction occurs at the level of the receptor itself: although Met^tg^ is fully phosphorylated on Tyr_1234–1235_ that are critical for its kinase activation, it only becomes fully signalling competent after HGF stimulation, as shown by HGF-induced increased phosphorylation levels on Tyr_1003_ and Tyr_1349_ implicated in receptor signalling and endocytosis and by activation of Akt, STAT3, and ERKs. Thus, in *Del-R26* ^*Met*^ cells, HGF switches Met from a subthreshold signalling status into a fully signalling competent form. These results also show that the *R26* ^*stopMet*^ mice represent a valuable genetic model to explore the consequences of moderate perturbations of Met as its full signalling competence still depends on ligand stimulation. A second point of restriction occurs at the levels of Gab1, which is phosphorylated at comparable levels in control and *Del-R26* ^*Met*^ cells upon HGF stimulation. Notably, in the absence of HGF stimulation Gab1 is phosphorylated in *Del-R26* ^*Met*^ hepatocytes (also expressing endogenous Met), but not in *Del-R26* ^*Met*^ mesenchymal cells (not expressing endogenous Met), indicating that the competence of cells to restrict signalling of enhanced Met is different according to the cell type, and that this competence can be influenced by factors such as expression levels of the receptor. Another restriction occurs further downstream, as revealed by the comparable phosphorylation levels of Akt, STAT3, and ERKs observed in control and *Del-R26* ^*Met*^ hepatocytes (by biochemical studies in culture) and myoblasts (by IHC in vivo). Such restrictions imply that different cell types possess distinct mechanisms that sense and calibrate the level of activation required for and compatible with biological programs. Overall, our studies provide additional insights into the existence of an exquisite monitoring of signalling levels within cells to attenuate enhanced RTK levels.

For cells that do not express endogenous Met, the issue of buffering an ectopic Met is only relevant provided that cells possess the adequate co-factors and downstream effectors. When they do, buffering may occur through mechanisms similar to those in cells with endogenous Met. Whereas most territories/cell types with ectopic Met appear capable of resilience, the limb mesenchyme is vulnerable to this signalling perturbation, illustrated by changes in gene expression, by failure of HGF bioavailability, and by resulting myoblast migration defects.

### Conditional *R26* ^*stopMet*^ mouse model to enhance wild-type Met signalling in skeletal muscle

The conditional *R26* ^*stopMet*^ mouse model represents a valuable tool for future studies on whether and how enhanced Met signalling impacts muscle development at later time points and during regeneration [55]. This issue is consistent with the persistence of Met expression in migratory muscles even after completion of the migration process, as shown by *Met* ^*LacZ/+*^ embryos at E13.5 (S8 Fig). The migration defect we report here may be clinically relevant as failure of subsets of myoblasts to migrate properly might represent a developmental dysfunction at the root of some muscle pathologies. This link is nicely illustrated by our recent findings showing that alterations of *Fat1* are associated with Facioscapulohumeral dystrophy (FSHD) [56]. Loss of *Fat1* in mice causes muscle shape defects resulting from altered migration polarity of a selective group of muscles matching those affected in FSHD [56]. *Fat1* expression is also regulated by HGF/Met, by Pax3-FKHR, and by Lbx1 [42, 45, 57]. Alteration of Met signalling can also impact muscle homeostasis by causing atrophy [58] and tumour formation. For example, Met is overexpressed in rhabdomyosarcoma, where it contributes to invasive growth [57], and *Met* gene amplification is frequently associated with sarcoma susceptibility in muscular dystrophy mouse models [51]. Therefore, *R26* ^*stopMet*^ mice can be instrumental for future evaluation of how Met signalling impacts on muscle physiology, regeneration, and/or pathologies.

### Conclusion

The *R26* ^*stopMet*^ mice modelling conditional gain of RTK signalling exemplify robustness versus sensitiveness of cells to signalling perturbations in order to ensure reproducible developmental outcomes. The genetic approach we employed permits subtle changes in RTK signalling, in contrast to others where constitutive RTK activation, either by point mutations or ligand overexpression, leads to dramatic biological consequences. It is therefore not surprising that the *Del-R26* ^*Met*^ mice do not recapitulate defects reported in other transgenics with an over-activation of the HGF/Met system. It is the case of mice in which HGF over-expression causes aberrant myoblast and neural crest migration, leading to ectopic muscle formation and melanosis in the central nervous system [59, 60]. A number of transgenic mice have been instrumental to demonstrate the capability of oncogenic forms of RTKs to trigger neoplasia. However, these mice do not permit assessment of how cells perceive and handle moderate changes in RTK signalling overtime. The *R26* ^*stopMet*^ genetic setting represents therefore a suitable system to explore the in vivo robustness of cells to subtle increase of RTKs signalling during tissue homeostasis as well as during development and may disclose unexpected switches in cell sensitivity from development to adulthood.

## Materials and Methods

### Ethics statement

All procedures involving the use of animals were performed in accordance with the European Community Council Directive of 24 November 1986 on the protection of animals used for experimental purposes (86/609/EEC). The experimental protocols were carried out in compliance with institutional Ethical Committee guidelines for animal research (comité d’éthique pour l’expérimentation animale–Comité d’éthique de Marseille; agreement number D13-055-21 by the Direction départementale des services vétérinaires–Préfecture des Bouches du Rhône).

### Transgenic lines and genotype analysis

The generation of the *R26* ^*stopMet*^ mice (international nomenclature *Gt(ROSA)26Sor* ^*tm1(Actb-Met)Fmai*^) carrying a conditional mouse–human chimeric *Met* transgene into the *Rosa26* locus *was* previously reported [24, 25]. The mouse lines expressing Cre recombinase under *Prx1* [31] and *Pax3* [30] promoters, the *Deleter-Cre* [26], *Mlc3F* ^*2E*^ *-LacZ* transgenic [27], the *Met* ^*d(neo/+)*^ [18] and *Met* ^*LacZ/+*^ [22] mouse lines were previously described. Mice and embryos were genotyped by PCR analysis of genomic DNA as reported in the above studies. For embryo collection, embryonic days were counted considering midday post-coitum as E0.5. All procedures involving the use of animals were performed in accordance with the European Community Council Directive of 24 November 1986 on the protection of animals used for experimental purposes (86/609/EEC). The experimental protocols were carried out in compliance with institutional Ethical Committee guidelines for animal research (comité d’éthique pour l’expérimentation animale–Comité d’éthique de Marseille; agreement number D13-055-21 by the Direction départementale des services vétérinaires–Préfecture des Bouches du Rhône).

### RNA in situ hybridisation and histological analysis

For ISH, embryos were collected in phosphate buffered Saline (PBS) and fixed in 4% paraformaldehyde (PFA) overnight. Whole mount RNA ISH done by using the relevant digoxigenin-labeled RNA probes. X-Gal and Salmon-Gal staining were performed as previously described [23, 24, 61]. For IHC of P0 limbs, newborns were embedded in cold glycol methacrylate (Technovit 8100) for cryosections. Limbs were cut transversally (16μm) and processed for antibodies staining as described [24]. For IHC at E10.5, embryos were embedded in paraffin, cut transversally (10μm), and processed for antibodies staining as described [62].

### MDCK scattering assays

Procedures were performed as described [49, 50]. Briefly, MDCK cells (ATCC, Rockville, MD) were seeded in 96-well plates (for forelimb co-cultures; Corning, Acton, MA) or on coverslips (for forelimb mesenchymal cells co-cultures) at 1250 cells/cm^2^ in DMEM containing 10% (v/v) fetal bovine serum (FBS), 100U/mL penicillin, 100μg/mL streptomycin, 4mM L-glutamine, and 1mM of sodium pyruvate. Cells were first incubated for 24 h at 37°C, in 5% CO_2_ to allow attachment and colony formation. For forelimb co-cultures, plates were processed as followed. In the first group, the media was changed with fresh media containing increasing concentrations of recombinant human HGF (0.1, 0.3, 1, 3, 10, 30ng/ml; R&D) to estimate the bioactivity dose of HGF released by wild-type limbs (comparable to 5ng/ml of recombinant human HGF). In the second group, media was replaced and added together with freshly dissected 2 forelimbs derived from E10.5 embryos of different genotypes (done by PCR using remaining tissue). Twenty-four hours later, the MDCK cells were fixed with 4% PFA in PBS, then cells were stained with Crystal violet. For quantification, at least 10 images with several cell colonies were analysed. Three different categories were defined: category 1 corresponds to not-scattered colonies; category 2 corresponds to colonies in which cells start losing their contact; category 3 corresponds to colonies with visible scattered cells.

### Limb mesenchymal cell cultures

Forelimbs from E10.5 embryos were dissected in HBSS supplemented with 100U/mL penicillin, 100μg/mL streptomycin, and 7mM Hepes (Life Technologies) and incubated for 5 min in 2% trypsin (Sigma). Limbs from individual embryos were processed separately and a piece of the body was used for genotyping. Then, trypsin was inactivated with DMEM supplemented with 10% (v/v) FBS, 100U/mL penicillin, 100μg/mL streptomycin, 4mM L-glutamine, 1mM of sodium pyruvate, and non-essential amino acids (defined as complete media). Cells were dissociated by pipetting, then spin down for 5 min, resuspended in DMEM complete media, and plated in 4 well plates (2 forelimbs per well). Cells were grown at 37°C in a humidified atmosphere of 5% CO_2_. For biochemical analysis of conditioned media, after 48 hrs cells were washed twice then cultured in DMEM complete media without serum for 24 hrs. For scattering assays, after 48 hrs a coverslip with previously plated MDCK cells was placed upside down in order to have MDCK cells in contact with media pre-conditioned by limb mesenchymal cells. In order to avoid MDCK and limb mesenchymal cell contact, a spacer was added between the plate and the coverslip. For HGF/Met inhibition, Met chemical inhibitors (cryzotinib, PHA665752, and SU11274; 1μM; Selleckchem) or blocking anti-HGF antibody (30μg/ml; R&D) where added prior to co-culture with MDCK cells on coverslips. After 24 hrs co-cultures, coverslips were fixed and processed as described above.

### Western blots

Culture of primary embryonic hepatocytes were performed as previously described [28, 46–48]. Protein extracts from embryonic hepatocytes and limb mesenchymal cells were prepared and western blot (WB) analysis was performed as previously described [63, 64]. For biochemical analysis, conditioned media was collected, spin down to remove cellular debris, and then incubated with either heparin-beads or lectin-beads (Amersham).

### Antibodies

Antibodies used were from Cell Signaling: anti-Met 25H2 (1:2000 for WB), anti-phospho-Tyr_1234/1235_-Met (1:2000 for WB; 1:50 for IHC), anti-phospho-Tyr_1003_-Met (1:2000 for WB), anti-phospho-Tyr_1349_-Met (1:1000 for WB), anti-phospho-Tyr_627_-Gab1 (1:2000 for WB), anti-phospho-Ser_473_-Akt (1:2000 for WB; 1:20 for IHC), anti-phospho-Ser_727_-Stat3 (1:2000 for WB), anti-phospho-Thr_202_-Tyr_204_-ERKs (#9106; 1:10000 for WB), anti-phospho-Thr_202_-Tyr_204_-ERKs (#4376; 1:150 for IHC), anti-ERKs (1:10000 for WB); from Santa-Cruz Biotechnology: anti-mouse Met (1:200 for WB), anti-human Met (1:1000 for WB), anti-HGF (1:500 for WB); from Abcam: anti-β-galactosidase (1:2000 for WB); from R&D: anti-mouseHGF (1:50 for IHC); from Sigma-Aldrich: anti-actin (1:12000 for WB); from Assay Designs: anti-human Met (1:500 for IHC); from hybridoma bank: Pax3 (1:10 for IHC), anti-myosin heavy chain II (MF20; 1:50 for IHC); from Jackson: anti-rabbit IgG-peroxidase or anti-mouse IgG-peroxidase (1:4000 for WB), anti-mouse fluorescent-coupled secondary antibodies (1:400 for IHC), anti-mouse or rabbit biotin-coupled secondary antibodies (1:500 for IHC).

### Quantitative RT-PCR analysis

Total RNA was isolated from embryos using the RNeasy Mini Kit (Qiagen, Valencia, CA) according to manufacture instruction. cDNA was generated using the Reverse Transcription Kit (Biorad). cDNA (30ng) was amplified by real time PCR using 4 μL SYBR Green qPCR SuperMix-UDG with Rox (Biorad) and 2μL of forward and reverse primers (0.1 μM). The analysis was performed on each sample in triplicates using Applied Biosystems (Foster City, CA). Relative transcript levels were calculated using the comparative Ct method and normalized to the housekeeping gene GAPDH. Primer sequences are listed in S1 Table.

### Skeletal staining

New-born mice were scarified and placed in water overnight at 4°C, then eviscerated, and skin was removed. Samples were fixed in 96% ethanol overnight. Cartilage staining was performed using alcian blue solution for 24 hours (0,15mg/ml alcian blue from Sigma in 1:4 volumes of acetic acid glacial and 96% ethanol). Samples were then rinsed in ethanol 96% for 1 hour and clearing was done for 6 hours in 2% KOH. Bone staining was performed using alizarin red solution for 24 hours (0,07mg/ml alizarin red from Sigma in KOH 1%). Samples were treated with 1% KOH/20% glycerol, then stored in glycerol/ethanol (1:1 volume).

### Image processing and analyses

For whole mount ISH, quantification of signal intensity in limbs was performed using the ImageJ software. Briefly, images were first converted to a grey scale and then inverted to a negative scale (with the highest signal intensity matching being white and the lowest black). Signal intensity was measured along a horizontal line of a given pixel length (matching the forelimb). After background and threshold subtraction, the values were averaged between several samples of each genotype to generate an average signal distribution plot (considering left and right limbs separately). The total signal intensity was also calculated for each sample and plotted individually.

For Met^25H2^ and HGF protein levels, quantification of signal intensity was done on western blots using the image J software. Images were process as described above. Values were averaged between different samples.

### Statistical analysis

Results were expressed as the mean ±SEM. Statistical significant differences were estimated by applying unpaired t-Student test for data showing normal distribution and by Mann-Whitney test otherwise. P values are indicated in Figs.

## Supporting Information

S1 Fig Strategy to ubiquitously enhance wild-type Met in developing embryos. (A) Schematic representation of transgenic mice carrying the LacZ-stop cassette followed by chimeric Met before (*Rosa26* ^*LacZ-stop-Met*^, namely *R26* ^*stopMet*^) and after Cre-mediated recombination (*tissue-specific-R26* ^*Met*^). (B, C) Whole mount (B) or transverse section (C) β-galactosidase staining of E10.5 *R26* ^*stopMet*^ and *Del-R26* ^*Met*^ embryos. Note that different degree of Cre-mediated recombination results in *Del-R26* ^*Met*^ mutants with high (right) or low (middle) β-galactosidase activity. hb: hindbrain; ov: optic vesicles; sc: spinal cord; di: diencephalon. (D) Genotype analysis of embryos showing the mutant allele before and after Cre recombination. Note that the efficiency of Cre-mediated recombination results into *Del-R26* ^*Met*^ embryos with total (right) or partial (middle) deletion of the LacZ-stop cassette. Scale: 500μm.
(TIF)

S2 Fig Expression pattern of human *Met* and mouse *Met* in control and *Del-R26* ^*Met*^ embryos. Whole mount ISH with human *Met* (*hMet*) and mouse Met (*mMet*) probes in E10.5 control and *Del-R26* ^*Met*^ embryos. Scale: 500μm.
(TIF)

S3 Fig Immunohistochemical analysis of phospho-Met and transgenic Met in E16.5 control and *Del-R26* ^*Met*^ embryos. (A) In *Del-R26* ^*Met*^ embryos, whereas Met^tg^ (detected by human Met antibodies; hMet) is expressed by most cell types in all tissues, phospho-Met is present only in a restricted number of cell types. Middle and bottom panels show enlargement of top panels at the levels of different organs. (B) Schematic representation of E16.5 embryos showing the level of sections reported in panel A and C. (C) Immunohistochemical analysis of phospho-Met and human Met (hMet) in control embryos showing background levels. (D) Table summarizing organs positive or not for phospho-Met. Scale: 200μm.
(TIF)

S4 Fig Genetic analysis of muscle development using the *MLC-LacZ* transgenics. (A) Whole mount β-galactosidase staining showing reduced developing appendicular muscles in E13.5 *Del-R26* ^*Met*^ embryos compared to controls. Note residual cytoplasmic β-galactosidase staining in the *R26* ^*stopMet*^ line due to a small proportion of cells in which the LacZ-stop cassette was not completely deleted. (B) Schematic representation of transgenic mice carrying the *MLC-LacZ* transgene alone (controls) or together with the transgenic Met in *Del-R26* ^*Met*^ embryos (limbs are outlined in bottom panels). Scale: 500μm.
(TIF)

S5 Fig Genetic analysis of migrating myoblasts in *Prx1-R26* ^*Met*^ and *Pax3-R26* ^*Met*^ conditional mutants. (A, B) Whole mount ISH of embryos with *Pax3* probe showing reduced migrating myoblasts in the forelimbs of *Prx1-R26* ^*Met*^ (A), but not of *Pax3-R26* ^*Met*^ (B) embryos. As in *Pax3-Cre* line the *Cre* gene is inserted in the *Pax3* locus resulting also into a loss-of-functional allele, the adequate control embryos in panel B correspond to *Pax3-Cre* heterozygous (*Pax3*). (C, D) Quantification analyses of *Pax3* positive domain in forelimbs. Left panels: each plot represents the average signal distribution along the white line in forelimbs. Right panels: quantifications and statistical analyses of the sum of signal intensity based on intensity plots in left panels. Numbers of samples: control, n = 11; *Prx1-R26* ^*Met*^, n = 6; *Pax3*, n = 4; *Pax3-R26* ^*Met*^, n = 4. The sum of *Pax3* signal intensity was calculated between point A and B: A indicating a fixed position between the somites and the limb whereas B being placed at a fixed distance from A. Note the reduced *Pax3* level in *Prx1-R26* ^*Met*^ mutants. (E) Left panels: Whole mount ISH of E12.5 embryos with *MyoD* probe showing reduced developing appendicular muscles in *Prx1-R26* ^*Met*^ mutants (limbs are outlined in bottom panels). Right panel: *Prx1-R26* ^*Met*^ mice at birth show hyperflexed forelimbs. Scale: 500μm.
(TIF)

S6 Fig Ectopic Met in developing limbs does not cause major patterning defects. (A) Whole mount ISH of E10.5 control and *Del-R26* ^*Met*^ embryos with *Shh* (top) and *Fgf8* (bottom) probes. Note that *Shh* and *Fgf8* expression remain located in the posterior limb bud mesenchyme and in the apical ectodermal ridge, respectively. Scale: 500μm. (B) Skeletal staining of P0 control and *Del-R26* ^*Met*^ limbs showing no major patterning defects. Note that the deltoid tuberosity in the humerus is significantly reduced in *Del-R26* ^*Met*^ mutants (100%: n = 7). As the deltoid tuberosity requires muscle mechanical forces at later stages besides initiating signals such as BMP4 [65], it is likely that this defect is a consequence of lack of muscles rather than a phenotype caused by ectopic Met in limb mesenchyme. Scale: 2mm.
(TIF)

S7 Fig qRT-PCR analyses on the expression levels of candidate genes in control and *Del-R26* ^*Met*^ embryos. (A) qRT-PCR analysis of human Met (*hMet*) and *LacZ* transcripts in E10.5 control (n = 11) and *Del-R26* ^*Met*^ (n = 11) limbs. Each dots corresponds to transcript levels in forelimbs of E10.5 individual embryos (done in triplicate). Columns correspond to the average value, expressed as mean. Note that upregulation of *Met* ^*tg*^ (*hMet)* is paralleled by downregulation of the *LacZ* transcripts in *Del-R26* ^*Met*^ embryos. (B, C) qRT-PCR analysis of candidate genes in E10.5 control and *Del-R26* ^*Met*^ mutant limbs. No significant changes are observed. *Hs2st1*: heparan sulphate 2-O sulfotransferase 1. *Hs3st1*: heparan sulphate 3-O sulfotransferase 1. *Sdf1*: stromal cell-derived factor 1. Columns correspond to the average value, expressed as mean ± s.e.m. Mann-Whitney and Student-*t* test.
(TIF)

S8 Fig Met expression as visualized by the knock-in of the lacZ reporter in the *Met* locus. (A, B) Whole mount β-galactosidase staining showing *Met* distribution in developing muscles of E10.5 (A) and E13.5 (B) *Met* ^*LacZ/+*^ and *Met* ^*LacZ/d(neo)*^ embryos. Note: a) the absence of muscles in limbs of E10.5 and E13.5 *Met* ^*LacZ/d(neo)*^ embryos; b) that *Met* expression is restricted to a subgroups of developing muscles at E13.5; c) that developing limb tendons express also *Met* at E13.5 and the pattern of expression is not altered in *Met* ^*LacZ/d(neo)*^ mutants. Scale: 500μm.
(TIF)

S9 Fig Ectopic Met in limb mesenchyme does not affect HGF levels in conditioned media from limb mesenchymal cells, but alters HGF bioavailability. (A) Western blot analysis of conditioned media from control and *Del-R26* ^*Met*^ limb mesenchymal cells after enrichment with heparin (left) or lectin (right) beads. Note no major differences in the level of uncleaved HGF between control and mutants. Numbers on the top of control and mutant lanes correspond to quantification analyses of uncleaved HGF. Quantification of the processed form of HGF was not performed because of the presence of a nonspecific band with the same molecular weight (ns). Lanes with human recombinant HGF (rec-hHGF: 5ng) and non-conditioned media (media) after purification. (B) Quantitative analysis of MDCK cell scattering using co-cultures with dissected forelimbs from control and *Del-R26* ^*Met*^ embryos with a recombination efficiency higher or lower than 60%. Quantification was performed according to categories defined in Fig 9B. Note a significant increase of cell colonies corresponding to the 1^st^ category using forelimbs from highly recombined *Del-R26* ^*Met*^ mutants (control: n = 14; *Del-R26* ^*Met*^ >60% recombination: n = 5; *Del-R26* ^*Met*^ <60% recombination: n = 3). Mann-Whitney and Student-*t* test.
(TIF)

S1 Table Primer sequences used for qRT-PCR analyses. (DOCX)
