# Supplementary figures and images for: Tissue-Specific Gain of RTK Signalling Uncovers Selective Cell Vulnerability during Embryogenesis
Source: PLoS Genet. 2015 Sep 22;11(9):e1005533. doi: 10.1371/journal.pgen.1005533 (PMC4579069; doi:10.1371/journal.pgen.1005533)

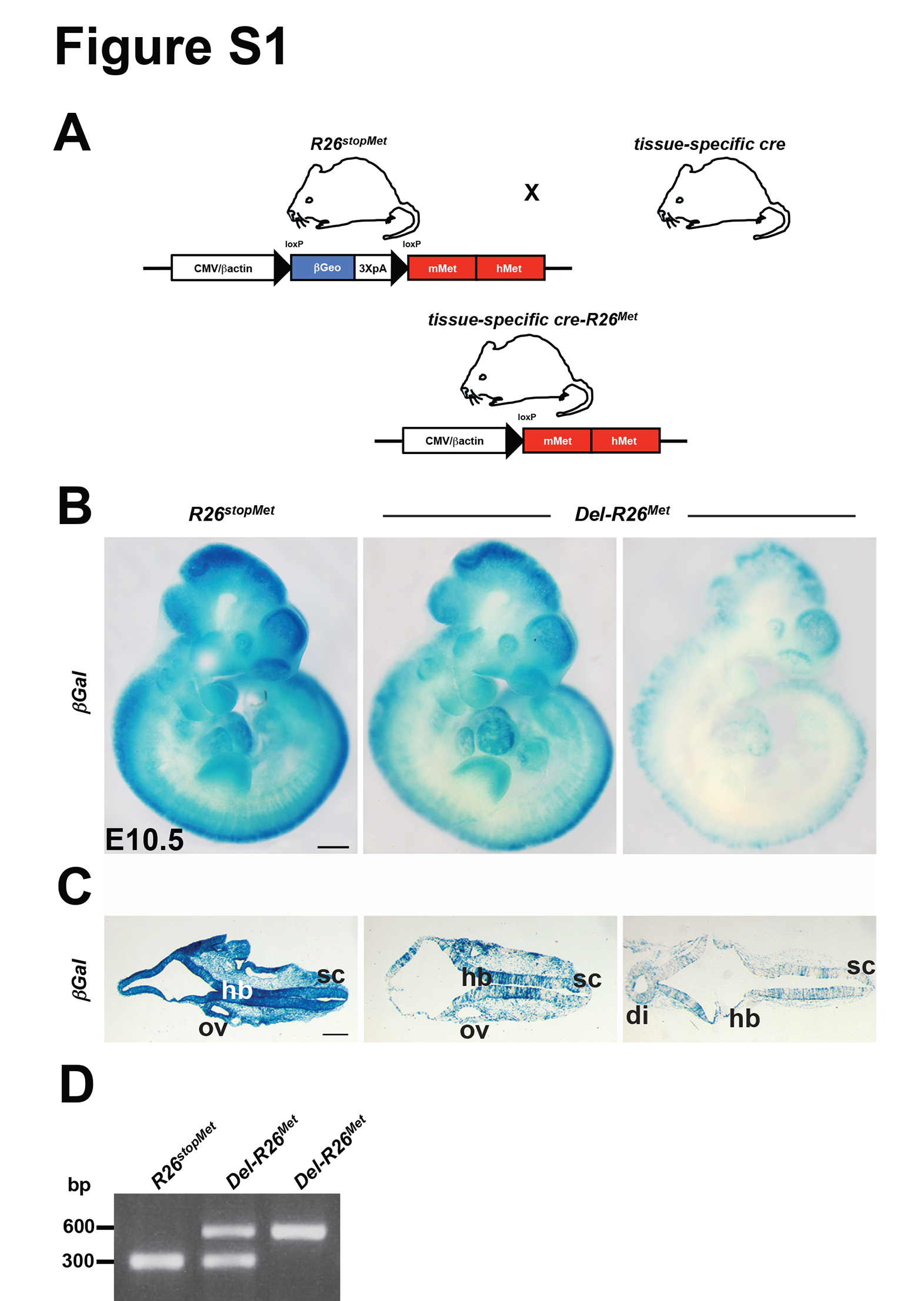

Supplement: S1 Fig — (A) Schematic representation of transgenic mice carrying the LacZ-stop cassette followed by chimeric Met before (Rosa26 LacZ-stop-Met, namely R26 stopMet) and after Cre-mediated recombination (tissue-specific-R26 Met). (B, C) Whole mount (B) or transverse section (C) β-galactosidase staining of E10.5 R26 stopMet and Del-R26 Met embryos. Note that different degree of Cre-mediated recombination results in Del-R26 Met mutants with high (right) or low (middle) β-galactosidase activity. hb: hindbrain; ov: optic vesicles; sc: spinal cord; di: diencephalon. (D) Genotype analysis of embryos showing the mutant allele before and after Cre recombination. Note that the efficiency of Cre-mediated recombination results into Del-R26 Met embryos with total (right) or partial (middle) deletion of the LacZ-stop cassette. Scale: 500μm. (TIF) [file pgen.1005533.s001.tif]

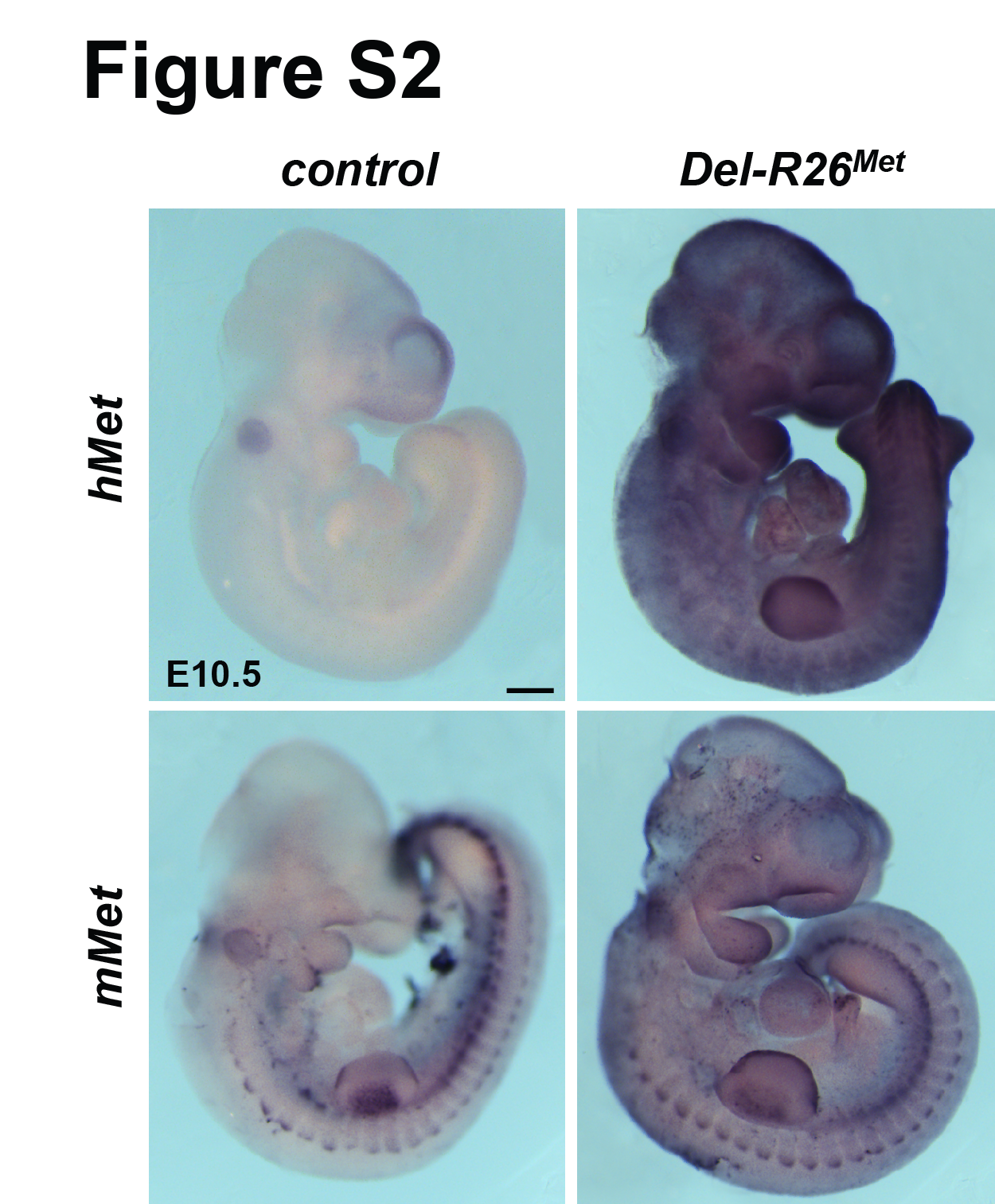

Supplement: S2 Fig — Whole mount ISH with human Met (hMet) and mouse Met (mMet) probes in E10.5 control and Del-R26 Met embryos. Scale: 500μm. (TIF) [file pgen.1005533.s002.tif]

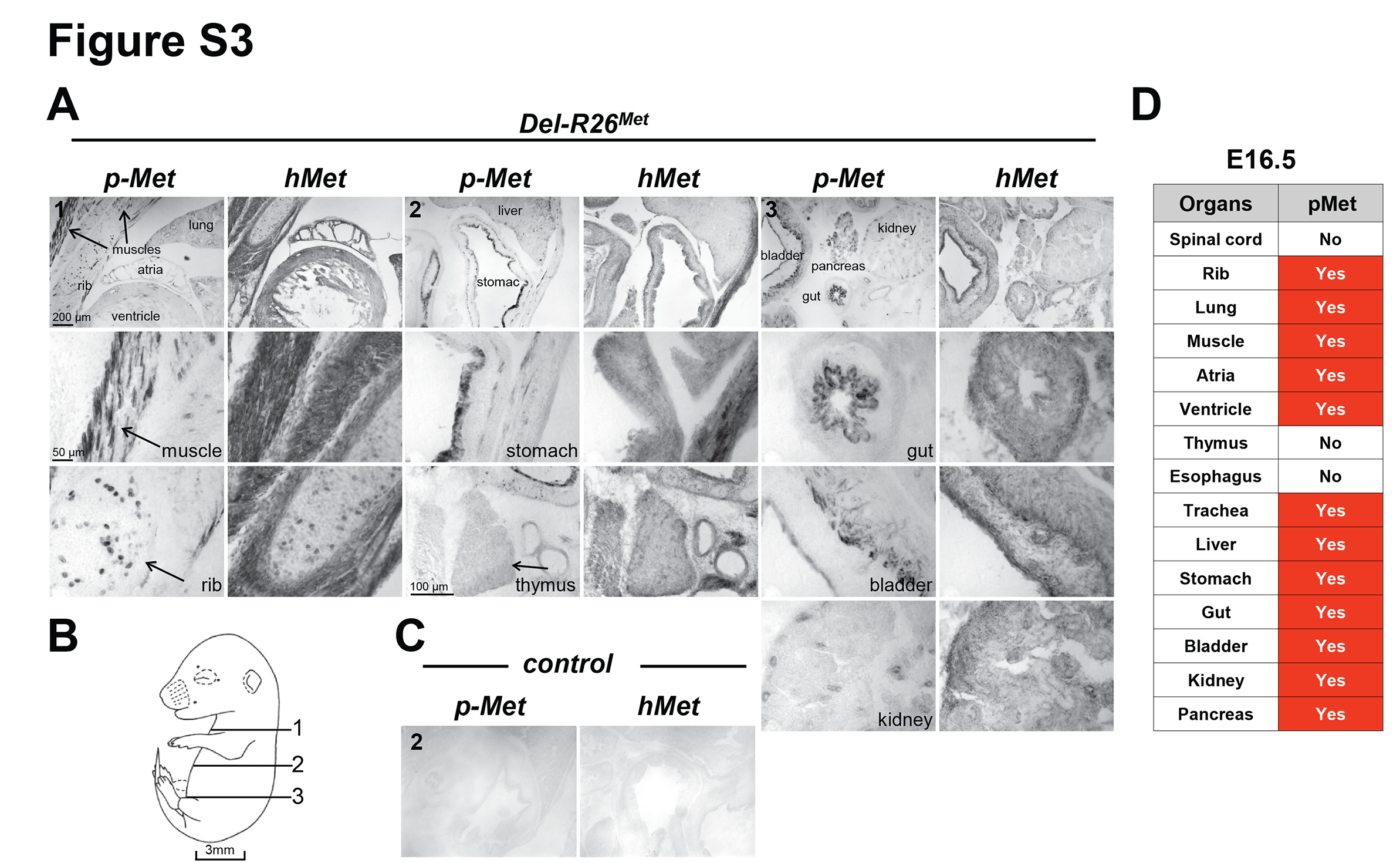

Supplement: S3 Fig — (A) In Del-R26 Met embryos, whereas Mettg (detected by human Met antibodies; hMet) is expressed by most cell types in all tissues, phospho-Met is present only in a restricted number of cell types. Middle and bottom panels show enlargement of top panels at the levels of different organs. (B) Schematic representation of E16.5 embryos showing the level of sections reported in panel A and C. (C) Immunohistochemical analysis of phospho-Met and human Met (hMet) in control embryos showing background levels. (D) Table summarizing organs positive or not for phospho-Met. Scale: 200μm. (TIF) [file pgen.1005533.s003.tif]

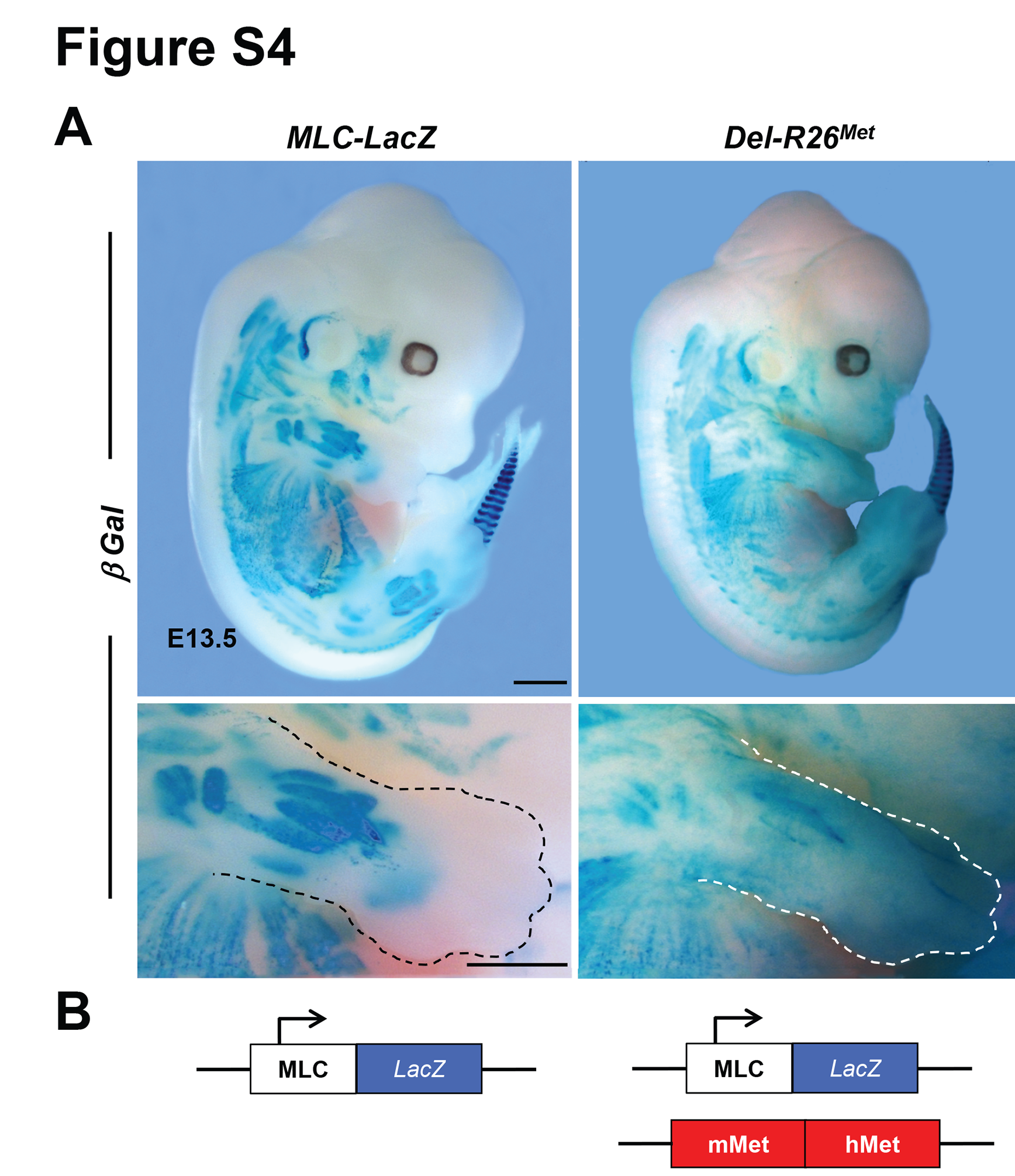

Supplement: S4 Fig — (A) Whole mount β-galactosidase staining showing reduced developing appendicular muscles in E13.5 Del-R26 Met embryos compared to controls. Note residual cytoplasmic β-galactosidase staining in the R26 stopMet line due to a small proportion of cells in which the LacZ-stop cassette was not completely deleted. (B) Schematic representation of transgenic mice carrying the MLC-LacZ transgene alone (controls) or together with the transgenic Met in Del-R26 Met embryos (limbs are outlined in bottom panels). Scale: 500μm. (TIF) [file pgen.1005533.s004.tif]

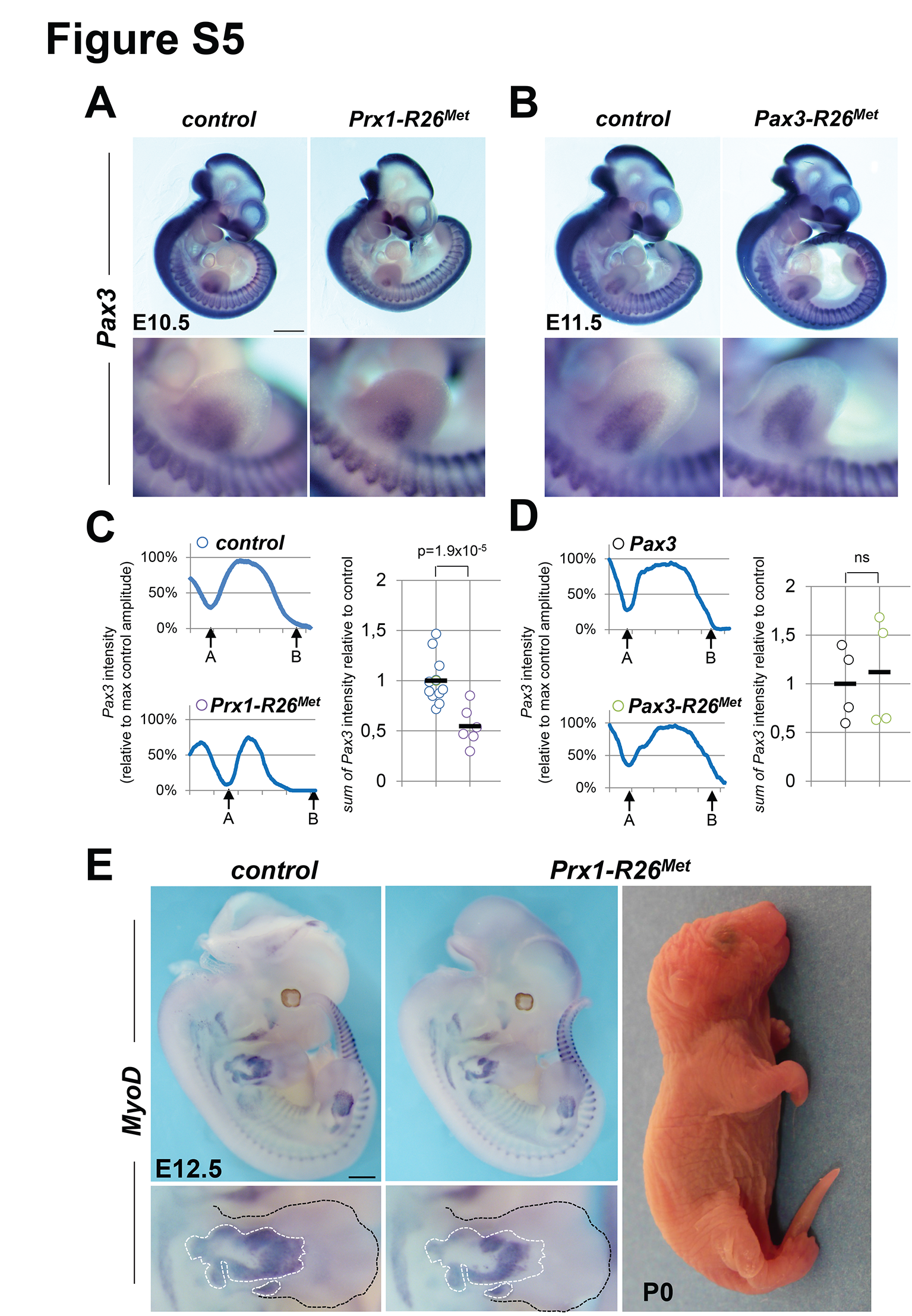

Supplement: S5 Fig — (A, B) Whole mount ISH of embryos with Pax3 probe showing reduced migrating myoblasts in the forelimbs of Prx1-R26 Met (A), but not of Pax3-R26 Met (B) embryos. As in Pax3-Cre line the Cre gene is inserted in the Pax3 locus resulting also into a loss-of-functional allele, the adequate control embryos in panel B correspond to Pax3-Cre heterozygous (Pax3). (C, D) Quantification analyses of Pax3 positive domain in forelimbs. Left panels: each plot represents the average signal distribution along the white line in forelimbs. Right panels: quantifications and statistical analyses of the sum of signal intensity based on intensity plots in left panels. Numbers of samples: control, n = 11; Prx1-R26 Met, n = 6; Pax3, n = 4; Pax3-R26 Met, n = 4. The sum of Pax3 signal intensity was calculated between point A and B: A indicating a fixed position between the somites and the limb whereas B being placed at a fixed distance from A. Note the reduced Pax3 level in Prx1-R26 Met mutants. (E) Left panels: Whole mount ISH of E12.5 embryos with MyoD probe showing reduced developing appendicular muscles in Prx1-R26 Met mutants (limbs are outlined in bottom panels). Right panel: Prx1-R26 Met mice at birth show hyperflexed forelimbs. Scale: 500μm. (TIF) [file pgen.1005533.s005.tif]

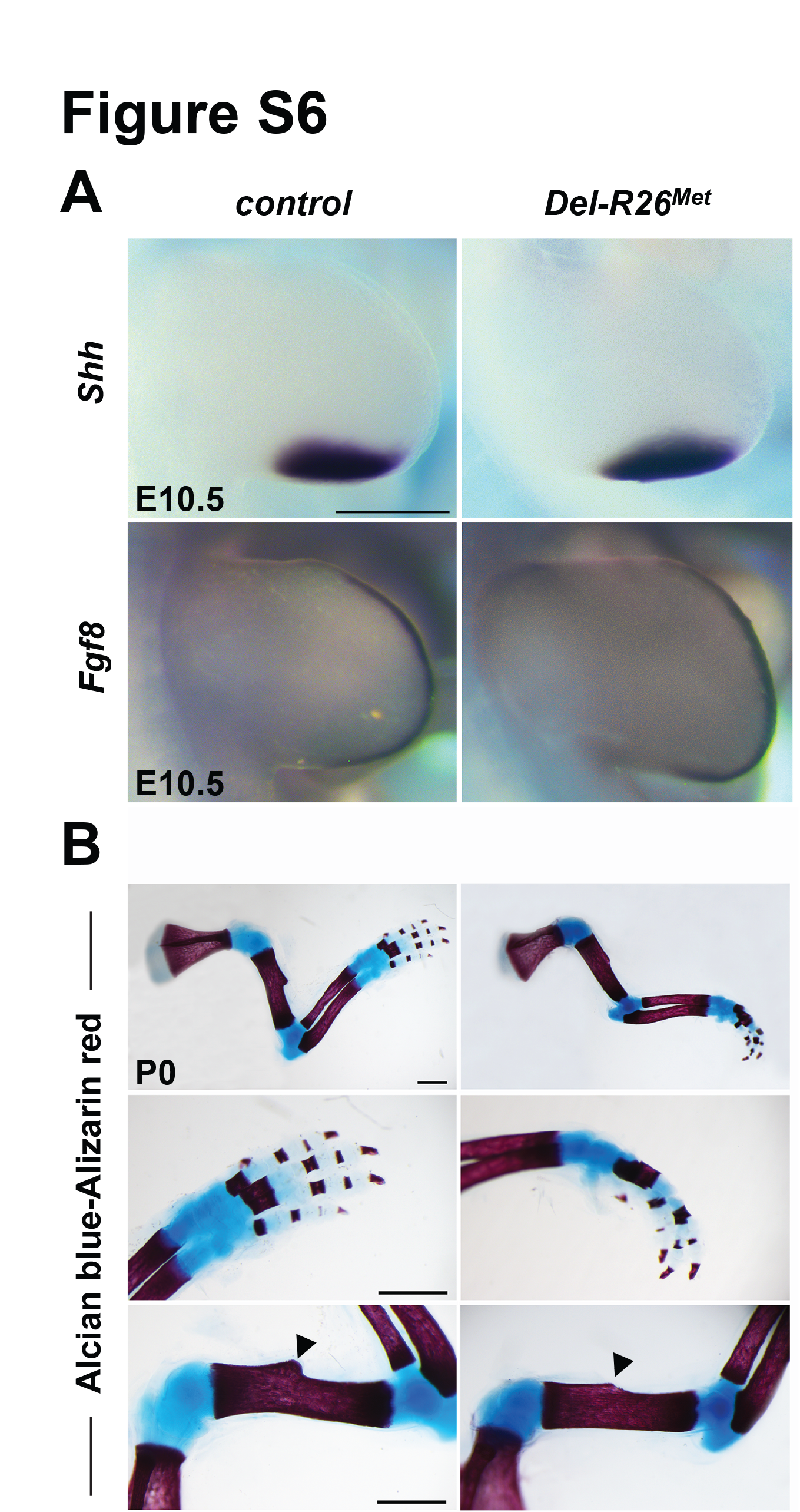

Supplement: S6 Fig — (A) Whole mount ISH of E10.5 control and Del-R26 Met embryos with Shh (top) and Fgf8 (bottom) probes. Note that Shh and Fgf8 expression remain located in the posterior limb bud mesenchyme and in the apical ectodermal ridge, respectively. Scale: 500μm. (B) Skeletal staining of P0 control and Del-R26 Met limbs showing no major patterning defects. Note that the deltoid tuberosity in the humerus is significantly reduced in Del-R26 Met mutants (100%: n = 7). As the deltoid tuberosity requires muscle mechanical forces at later stages besides initiating signals such as BMP4 [65], it is likely that this defect is a consequence of lack of muscles rather than a phenotype caused by ectopic Met in limb mesenchyme. Scale: 2mm. (TIF) [file pgen.1005533.s006.tif]

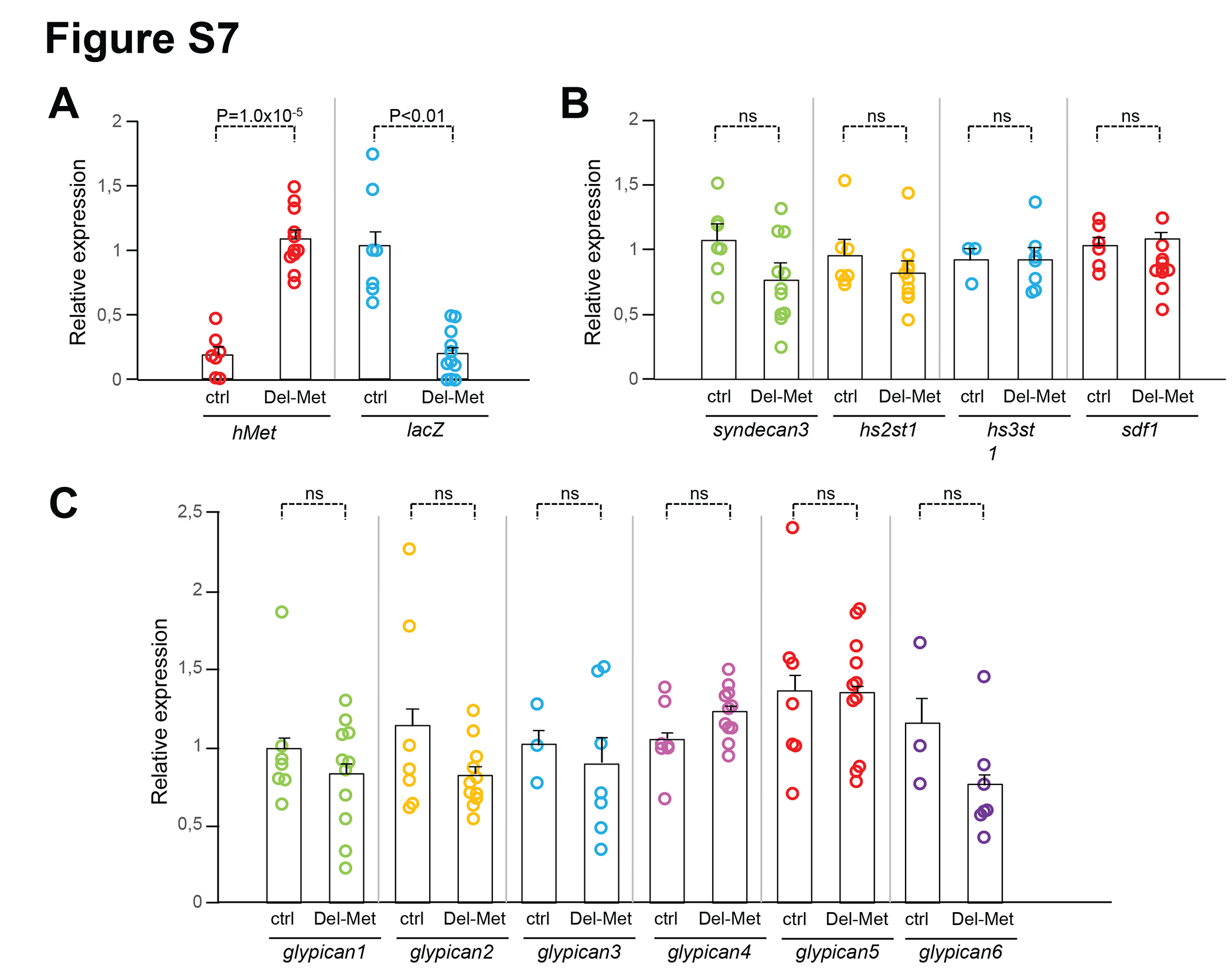

Supplement: S7 Fig — (A) qRT-PCR analysis of human Met (hMet) and LacZ transcripts in E10.5 control (n = 11) and Del-R26 Met (n = 11) limbs. Each dots corresponds to transcript levels in forelimbs of E10.5 individual embryos (done in triplicate). Columns correspond to the average value, expressed as mean. Note that upregulation of Met tg (hMet) is paralleled by downregulation of the LacZ transcripts in Del-R26 Met embryos. (B, C) qRT-PCR analysis of candidate genes in E10.5 control and Del-R26 Met mutant limbs. No significant changes are observed. Hs2st1: heparan sulphate 2-O sulfotransferase 1. Hs3st1: heparan sulphate 3-O sulfotransferase 1. Sdf1: stromal cell-derived factor 1. Columns correspond to the average value, expressed as mean ± s.e.m. Mann-Whitney and Student-t test. (TIF) [file pgen.1005533.s007.tif]

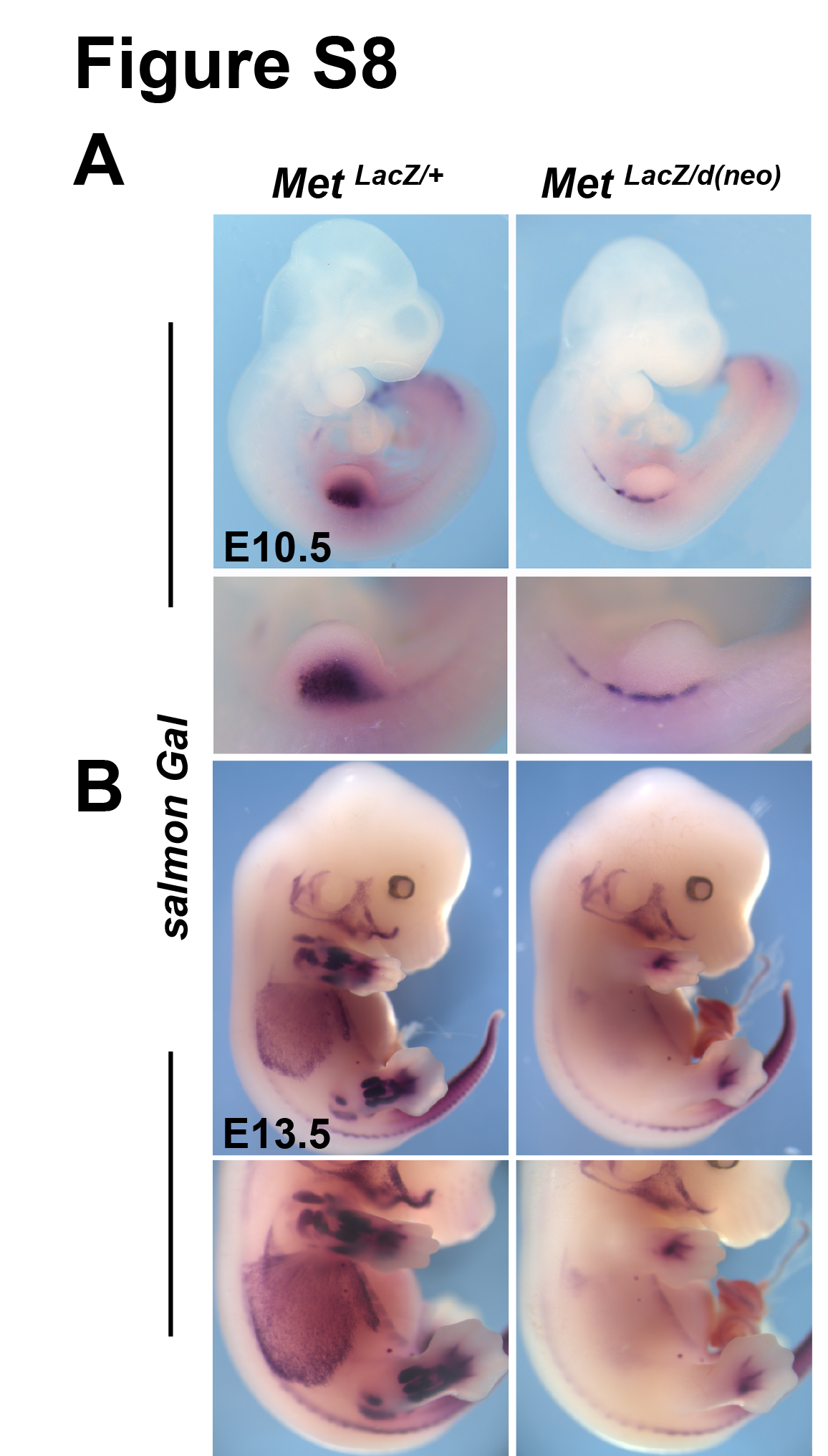

Supplement: S8 Fig — (A, B) Whole mount β-galactosidase staining showing Met distribution in developing muscles of E10.5 (A) and E13.5 (B) Met LacZ/+ and Met LacZ/d(neo) embryos. Note: a) the absence of muscles in limbs of E10.5 and E13.5 Met LacZ/d(neo) embryos; b) that Met expression is restricted to a subgroups of developing muscles at E13.5; c) that developing limb tendons express also Met at E13.5 and the pattern of expression is not altered in Met LacZ/d(neo) mutants. Scale: 500μm. (TIF) [file pgen.1005533.s008.tif]

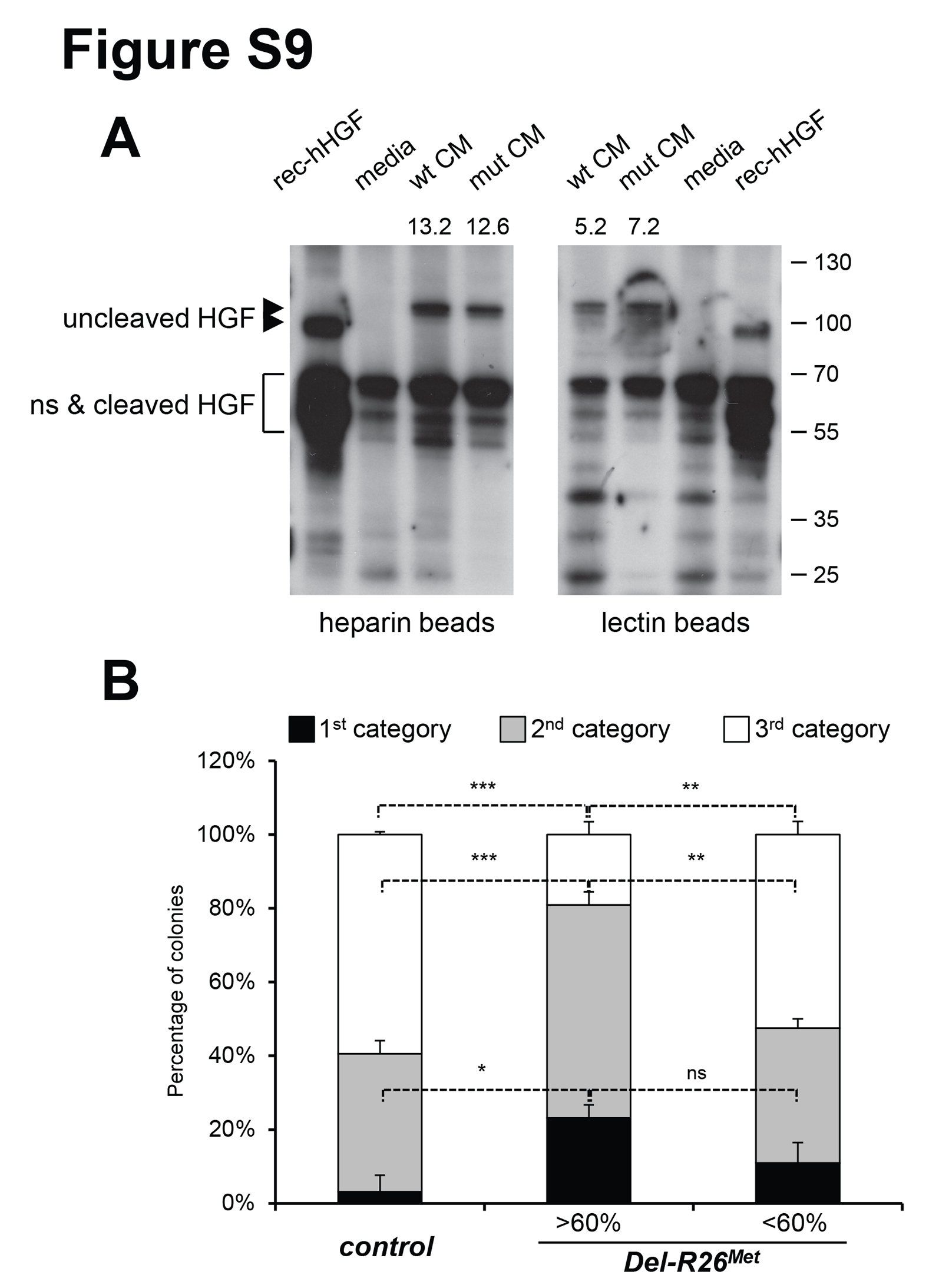

Supplement: S9 Fig — (A) Western blot analysis of conditioned media from control and Del-R26 Met limb mesenchymal cells after enrichment with heparin (left) or lectin (right) beads. Note no major differences in the level of uncleaved HGF between control and mutants. Numbers on the top of control and mutant lanes correspond to quantification analyses of uncleaved HGF. Quantification of the processed form of HGF was not performed because of the presence of a nonspecific band with the same molecular weight (ns). Lanes with human recombinant HGF (rec-hHGF: 5ng) and non-conditioned media (media) after purification. (B) Quantitative analysis of MDCK cell scattering using co-cultures with dissected forelimbs from control and Del-R26 Met embryos with a recombination efficiency higher or lower than 60%. Quantification was performed according to categories defined in Fig 9B. Note a significant increase of cell colonies corresponding to the 1st category using forelimbs from highly recombined Del-R26 Met mutants (control: n = 14; Del-R26 Met >60% recombination: n = 5; Del-R26 Met <60% recombination: n = 3). Mann-Whitney and Student-t test. (TIF) [file pgen.1005533.s009.tif]
