# Supplementary material for: Tissue-Specific Gain of RTK Signalling Uncovers Selective Cell Vulnerability during Embryogenesis
Source: PLoS Genet. 2015 Sep 22;11(9):e1005533. doi: 10.1371/journal.pgen.1005533 (PMC4579069; doi:10.1371/journal.pgen.1005533)
Supplement: S1 Table — (DOCX) [file pgen.1005533.s010.docx]

**Table S1. Primer sequences of genes analysed by RT-qPCR.**

| Gene | Forward primer | Reverse primer |
| --- | --- | --- |
| *Gpc1* | GCCGAAATGTGCTCAAAGGCTG | CCCAGAACTTGTCAGTGATGAGC |
| *Gpc2* | GGAGTTCCTTCACTTATGCCCTG | GTCCTTGGAGTTTCTCAGCCAG |
| *Gpc3* | CTGTGCTGGAACGGACAAGAAC | GTCAATGATCTGGCTAACCACCG |
| *Gpc4* | GAGGATGACTGCTGGAATGGCA | GACGAAGGATCAGTATGTCTGGC |
| *Gpc5* | GTGCTCCTGAACTTCCACTTGC | GGCTCTGTGTTGGTGTTCTCAC |
| *Gpc6* | AGAGGTTGCCAACCGAGTTTCC | TGCAAGGTCTCACAGTGGGCAA |
| *Sdc3* | AGGAGCCTGATGTTGCTGAGAG | CTCATCCTGGATGGTGGTCAGA |
| *Sdc4* | ATGCTGGCGGCTCGGATGACT | GGGCTCAATCACTTCAGGGAAG |
| *Notum* | CGTGGTACACTCAAGGATGTGC | GCCTTATGGCTGTCATGGAAGC |
| *Hs2st1* | ACCACCTGGAACGAGATGAAGC | ACTAGCCTCTCAATAGGGTCCC |
| *Hs3st1* | AGTGCCTGAGAGAATCCACAGC | GGCTTGTGCTTCTGAAGGTGGT |
| *Sdf1* | GGAGGATAGATGTGCTCTGGAAC | AGTGAGGATGGAGACCGTGGTG |
| *mMet* | GGCACTCTGGAGCTGTGTTG | CTCTTTCTCATCCACAGGAAGAG |
| *hMet* | CTGAAGCCGTTTTATGCACG | GCCACAGGAAAAACCCAAGTAG |
| *LacZ* | ATGGATGAGCAGACGATGG | CGGCGTTAAAGTTGTTCT |
| *Pax3* | GCGTCTCTAAGATCCTGTGCAG | GATTTCCCAGCTAAACATGCCCG |
| *Gapdh* | GTCTCCTGCGACTTCAACAGC | ACCACCCTGTTGCTGTAGCCGT |
